# A facile, rapid, one-pot regio/stereoselective synthesis of 2-iminothiazolidin-4-ones under solvent/scavenger-free conditions

**DOI:** 10.3762/bjoc.9.78

**Published:** 2013-04-10

**Authors:** Murugan Sathishkumar, Sangaraiah Nagarajan, Poovan Shanmugavelan, Murugan Dinesh, Alagusundaram Ponnuswamy

**Affiliations:** 1 Department of Organic Chemistry, School of Chemistry, Madurai Kamaraj University, Madurai 625021, Tamilnadu, India

**Keywords:** 2-iminothiazolidin-4-ones, regioselective, stereoselective, solvent-free, scavenger-free

## Abstract

A rapid and efficient one pot solvent/scavenger**-**free protocol for the synthesis of 2-iminothiazolidin-4-ones has been developed. Interestingly, the regio/stereoselective synthesis affords the regioisomeric (*Z*)-3-alkyl/aryl-2-(2-phenylcyclohex-2-enylimino)thiazolidin-4-one as the sole product in good yield. The selectivities observed have been rationalized based on the relative magnitude of the allylic strains developed during the course of the reaction. This is the first report wherein the impact of allylic strains in directing the regiocyclization has been noted.

## Introduction

**Figure 1:**
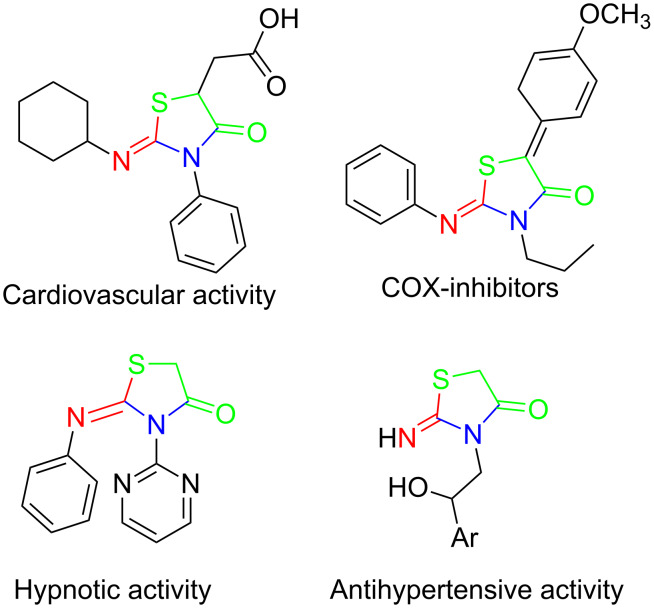
Medicinally relevant 2-iminothiazolidin-4-ones.

Thiazolidin-4-one derivatives are well known for their bioactivities such as antidiabetic [1], anticancer [2], calcium-channel blocker [3–4], platelet activating factor (PAF) antagonist [5] and anti-HIV [6] activity. In addition, 2-iminothiazolidin-4-ones exhibit remarkable hypnotic [7–8], antitubercular [9], cardiovascular [10] and cyclooxygenase (COX) inhibitory [11] activities (Figure 1).

A common strategy involved in the prevailing synthetic protocols for 2-iminothiazolidin-4-ones [12–14] is the cyclization of thioureas with α-halocarboxylic acids [15] or acyl halides [16–17] or carboxylic esters [18]. These protocols are generally solution phase methods using organic solvents and acid scavengers. In the present scenario, such protocols may not be recommended by the principles of green chemistry. Consequently, the search for simple and efficient environmentally friendly methodologies for the synthesis of 2-iminothiazolidin-4-ones is worth attempting.

In this regard, and in continuation of our recent reports on the solvent-free synthesis of amides [19–20], thioamides [21], cyclic imides [22], thiazolidin-4-ones [23], spirothiazolidin-4-ones [24], 1,2,3-triazoles [25], 1,2,3-triazolylchalcones [26], and 1,2,3-triazolyldihydropyrimidine-2-thiones [27], we herein present a one-pot solvent/scavenger-free synthetic protocol for 2-iminothiazolidin-4-ones. This environmentally benign method avoids toxic organic solvents and acid scavengers, the details of which are presented below.

## Results and Discussion

At the outset, optimization of the one-pot reaction was attempted by varying the solvents and using triethylamine as the acid scavenger (Table 1). The reaction was also attempted under solvent-free conditions. The latter was more promising in the sense that the reaction was very rapid affording the product **4f** in 15–20 min (Table 1, entries 7 and 8) compared to 2–6 h (Table 1, entries 1–6) in solvents. The structure of the product **4f** was assigned as (*Z*)-2-(2-phenylcyclohex-2-enylimino)-3-*p*-tolylthiazolidin-4-one based on the single-crystal XRD data [28–29] of its analogues (**4c** and **4j**).

**Table 1:** Optimization of solution-phase and solvent-free synthesis of 2-iminothiazolidin-4-one **4f**.

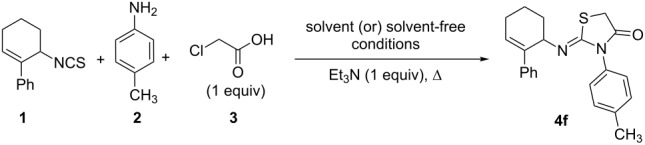				

Entry	Solvent	Temperature	Time	Yield (%)^a^

1	ethanol	reflux	4 h	52
2	acetonitrile	reflux	2 h	66
3	dioxane	reflux	4 h	46
4	THF	reflux	3 h	51
5	acetonitrile/ethanol (1:1)	reflux	3 h	49
6	DCM	reflux	6 h	41
7	solvent-free	80 °C	20 min	41
8	solvent-free	100 °C	15 min	54

^a^Yield of isolated product.

**Scheme 1:**
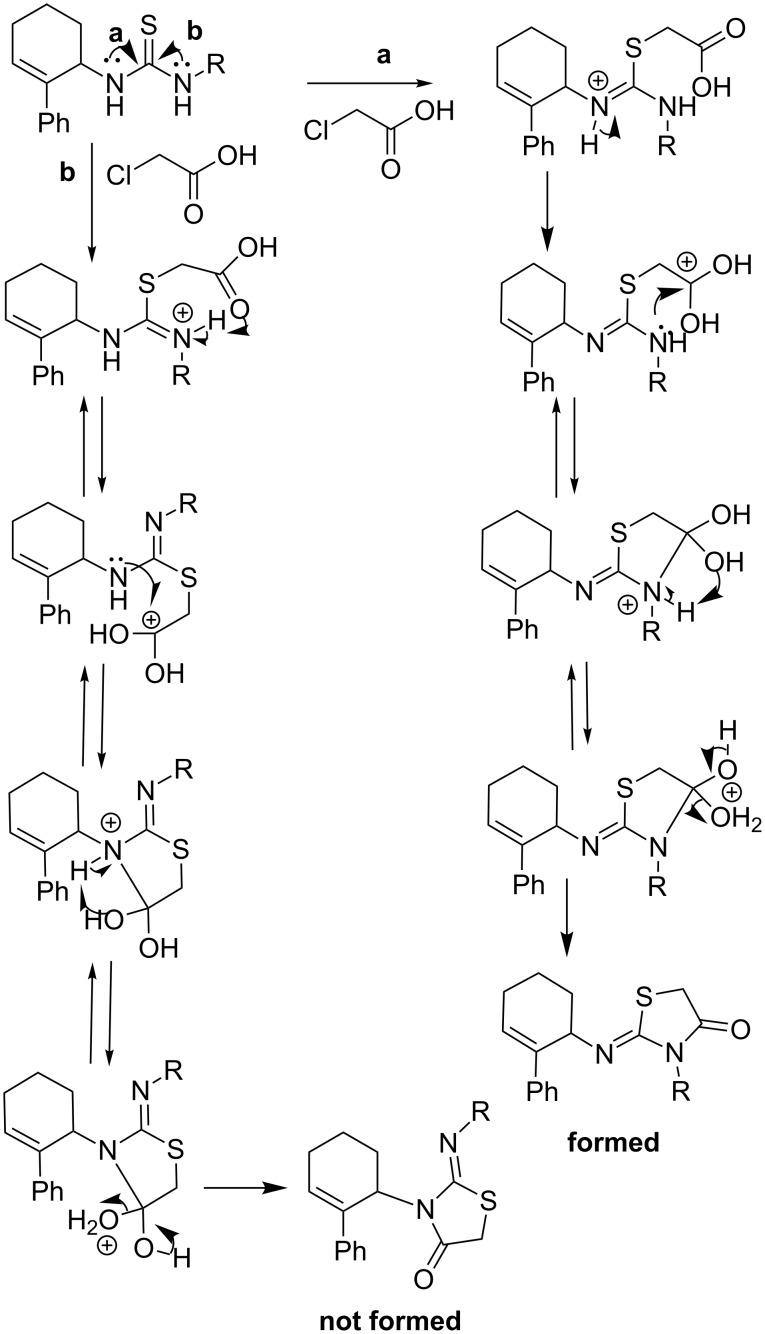
Plausible mechanism.

Though the rate of reaction rate could be accelerated, the yield of 2-iminothiazolidin-4-one **4f** was not good (41–66%) under both solution-phase and solvent-free conditions. Hence, as an attempt to optimize the yield, the solvent-free protocol was screened with and without the acid scavenger. Hereto, the yield of the product was poor (Table 2). Thus, the screening indicated that the scavengers had no positive, but rather an impeding effect. To develop an insight in this regard, a plausible mechanism of the reaction in the presence of the acid scavenger was proposed (Scheme 1).

From the mechanism, it can be envisaged that the acid scavenger may neutralize the HCl (that is generated during the course of the reaction) or the iminium ions by deprotonation. Also, another possibility is that the use of base as the scavenger may lead to the acid–base reaction resulting in the formation of the carboxylate anion of one the starting materials viz. chloroacetic acid or the thiourea-chloroacetic acid coupled product. This may retard the direct amine–carboxylic acid coupling, thus decreasing the yield of the product.

In view of the above perception, the solvent-free protocol was screened with one equivalent or in the absence of acid scavenger and varying equivalents of chloroacetic acid at 100 °C (Table 2). The optimum conditions were found to be with 3 equivalents of chloroacetic acid in the absence of acid scavenger affording a good yield of 2-iminothiazolidin-4-one **4f** (Table 2, entry 6).

**Table 2:** Screening of base and equivalents of chloroacetic acid in the solvent-free synthesis of 2-iminothiazolidin-4-one **4f**.

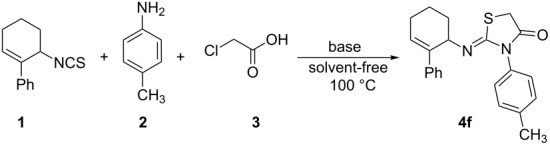				

Entry	Base	Base (equiv)	Chloroacetic acid (equiv)	Yield (%)^a^

1	Et_3_N	1	1	41
2	K_2_CO_3_	1	1	25
3	K_2_CO_3_	1	2	35
4	NaOH	1	2	24
5	pyridine	1	2	57
6	—	—	3	82

^a^Yield of the isolated product.

In this context, it is pertinent to mention that while the prevailing solution-phase protocol [30–32] uses an acid scavenger, such as sodium hydroxide, triethylamine, pyridine or sodium acetate, the solvent-free methodology involved in the present investigation does not require any acid scavenger.

**Figure 2:**
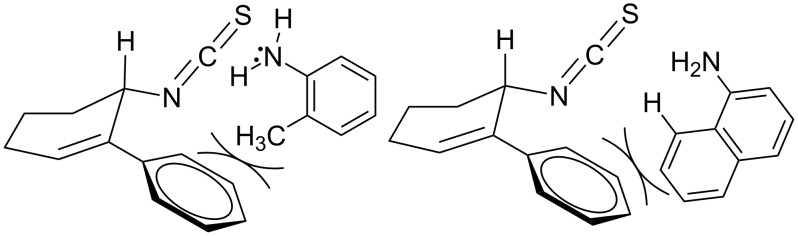
Retardation of the nucleophilic attack of amines on the isothiocyanate due to the steric effect.

The scope of the new synthetic protocol was proved through the synthesis of a library of 2-iminothiazolidin-4-ones (Table 3). However, its limitations were realized when the synthesis of *ortho*-tolyl/1-napthyl analogues and the para-substituted (NO_2_ and COOH) phenyl analogues failed. Apparently, the reason for this can be attributed to the retardation of the nucleophilic attack of the amines on the isothiocyanate due to the steric effect (Figure 2) in the former, and decrease of the nucleophilicity of the amines by the electron-withdrawing group in the latter, thus not affording the expected thiourea.

**Table 3:** Solvent/scavenger-free synthesis of 2-iminothiazolidin-4-ones **4a**–**n**.

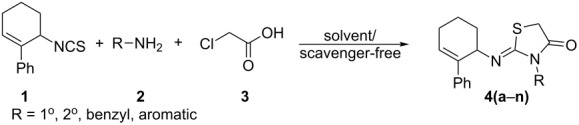			

Entry	Amine	Product	Yield (%)^a^

1	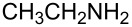	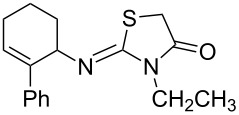 **4a**	79
2	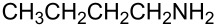	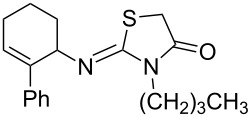 **4b**	84
3	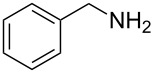	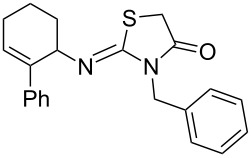 **4c**	87
4	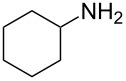	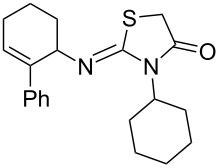 **4d**	84
5	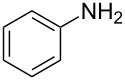	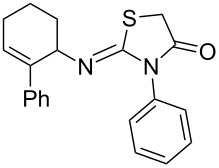 **4e**	80
6	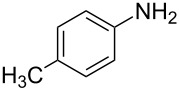	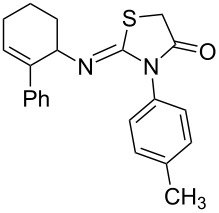 **4f**	82
7	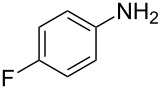	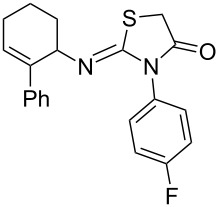 **4g**	80
8	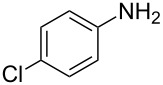	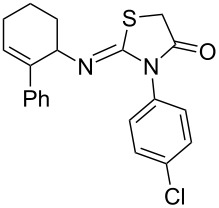 **4h**	83
9	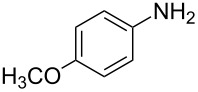	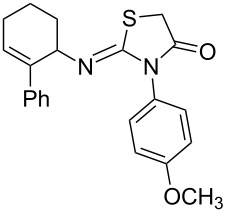 **4i**	85
10	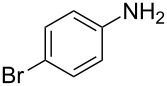	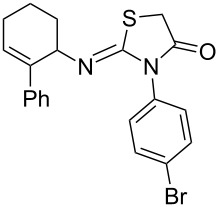 **4j**	81
11	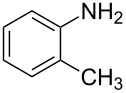	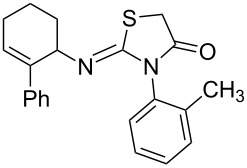 **4k**	—
12	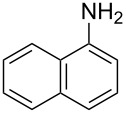	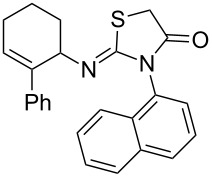 **4l**	—
13	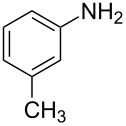	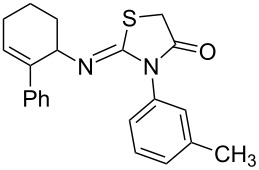 **4m**	trace
14	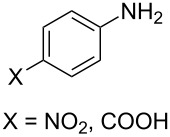	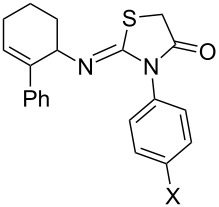 **4n**	—

^a^Yield of isolated product.

Having established the new protocol for the synthesis of 2-iminothiazolidin-4-ones, the method was extended to the rapid synthesis of a library of thiazolidinone derivatives (Table 4).

**Table 4:** Solvent/scavenger-free synthesis of thiazolidinone derivatives.

Entry	Thiourea	Thiazolidinone	Time (min)	Yield (%)^a^/Ref

1	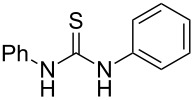	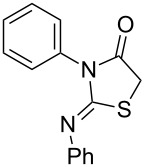	20	78/[17]
2^b^	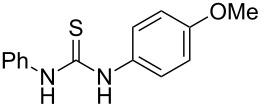	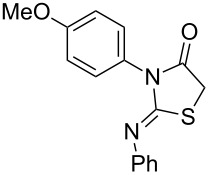	20	81/[17]
3^b^	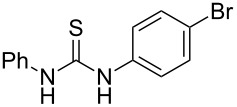	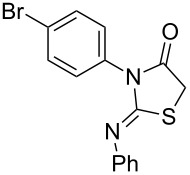	20	80/[17]
4^b^	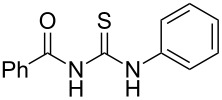	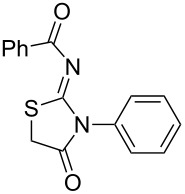	30	75/[16]
5^b^	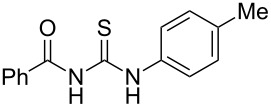	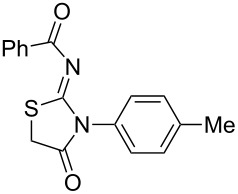	30	72/[16]
6	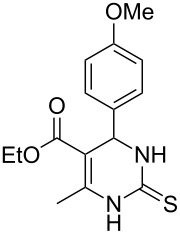	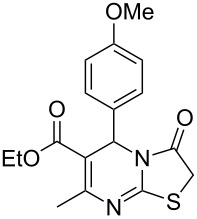	15	84/[33]
7	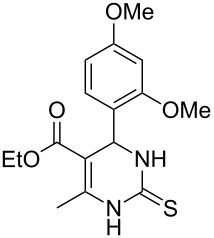	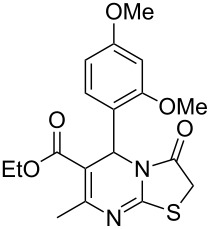	15	80/[33]
8	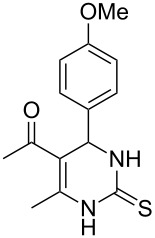	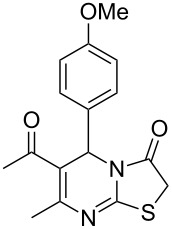	15	79/[33]
9	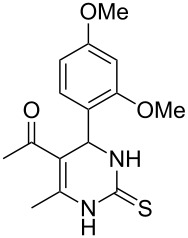	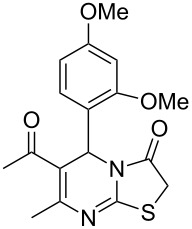	15	77/[33]
10	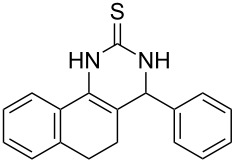	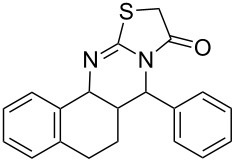	20	80/[34]
11	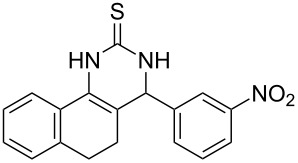	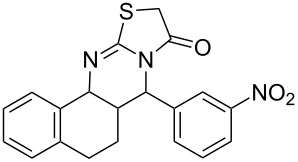	20	82/[34]
12	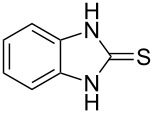	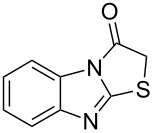	20	81/[32]

^a^Yield of isolated product, ^b^regioisomeric mixtures obtained.

**Figure 3:**
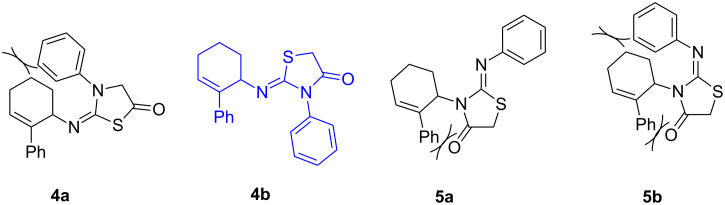
Possible regio/stereoisomeric products.

Further, it is pertinent to mention here the interesting regio/stereoselectivity noted in the synthesis. Though the formation of the four regio/stereoisomeric 2-iminothiazolidin-4-ones **4a**, **4b**, **5a** and **5b** is possible, it is novel to note that only one of them, viz. **4b**, is formed exclusively (Figure 3).

**Scheme 2:**
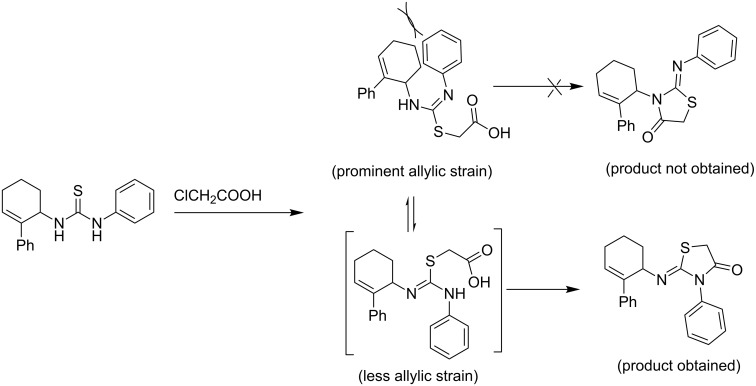
Regioselective cyclization in 2-iminothiazolidin-4-one synthesis directed by allylic strains.

The high regio/stereoselectivity of the reaction can be rationalized based on the relative magnitudes of allylic strains (A^1,2^ and A^1,3^) developed during the course of the regiocyclization (Scheme 2).

In this context, it is relevant to recall the literature reports on the factors directing the regioselectivity in the synthesis of 2-iminothiazolidin-4-ones. Only a couple of reports in this regard are available in the literature. While one of these reports suggests that the p*K*_a_ [17] of amines directs the regioselectivity, another investigation indicates that the chelating effect [18] of the substituent directs the regiochemical outcome. In both the reports, two regioisomeric 2-iminothiazolidin-4-ones are obtained. Thus, the present investigation affording a single regioisomeric product exclusively is the first report wherein the allylic strains are noted to direct the high regioselectivity.

**Figure 4:**
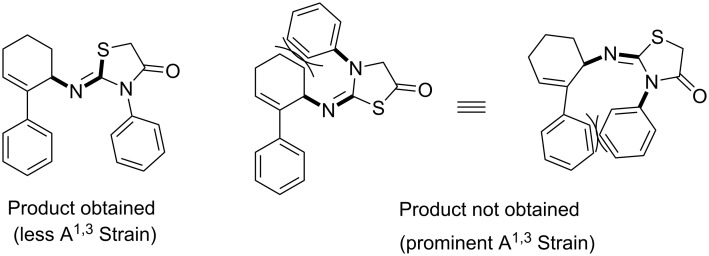
Stereoselectivity of the reaction directed by A^1,3^ strain.

Finally, the stereoselective formation of the (*Z*)-stereoisomer is also explicable based on allylic strain, which is summarized in Figure 4.

## Conclusion

In conclusion, a new solvent/scavenger-free synthetic protocol for 2-iminothiazolidin-4-ones has been reported. Unlike the prevailing solution-phase protocols employing organic solvents and acid scavengers, the present study avoids solvents and scavengers. The rate of the reaction is prominently enhanced under solvent-free conditions compared to that in the solution phase. Apparently, the intimacy of the highly polar reactants in the fused state in the absence of solvent may be responsible for the rate enhancement.

## Supporting Information

File 1 Experimental procedures and product characterization for compounds (**4a**–**j**).
