# Supplementary material for: A facile, rapid, one-pot regio/stereoselective synthesis of 2-iminothiazolidin-4-ones under solvent/scavenger-free conditions
Source: Beilstein J Org Chem. 2013 Apr 10;9:689–97. doi: 10.3762/bjoc.9.78 (PMC3628849; doi:10.3762/bjoc.9.78)
Supplement: File 1 — Experimental procedures and product characterization for compounds (4a–j). [file Beilstein_J_Org_Chem-09-689-s001.pdf]

Supporting Information

for

A facile, rapid, one-pot regio/steroselective synthesis of 2-iminothiazolidin-4-ones under solvent/scavenger-free conditions

Murugan Sathishkumar, Sangaraiah Nagarajan, Poovan Shanmugavelan, Murugan Dinesh and Alagusundaram Ponnuswamy*

Address: Department of Organic Chemistry, School of Chemistry, Madurai Kamaraj University, Madurai-625021, Tamilnadu, India.

Email: Alagusundaram Ponnuswamy* - ramradkrish@yahoo.co.in
* Corresponding author

Experimental procedures and product characterization for compounds 4a–j

Experimental section…………………………………………………… S2

General procedure for the synthesis of 2-iminothiazolidin-4-ones 4a–j S2–S6

Copies of ^{1}H and ^{13}C NMR spectra ……………………..……………… S7–S18

Experimental section

All chemicals, reagents and solvents were of commercial high-purity grade purchased from Avra Synthesis Pvt. Ltd. and Merck Ltd. India. Silica gel (60–120 mesh) was used for column chromatographic isolation and purification of the products. Melting points were measured in open capillary tubes and are uncorrected. Elemental analyses were performed on a Perkin Elmer 2400 Series II Elemental CHNS analyzer. The ^{1}H NMR, ^{13}C NMR, DEPT, H,H-COSY and C,H-COSY, HMBC were recorded on a Bruker (Avance) 300 MHz NMR instrument using TMS as an internal standard and $CDCl_3$ as a solvent. Chemical shifts are given in parts per million (δ-scale) and the coupling constants are given in hertz.

General procedure for the synthesis of 2-iminothiazolidin-4-ones (4a–j)

A mixture of 1-isothiocyanato-2-phenylcyclohex-2-ene (**1**, 0.5 g, 2.3 mmol) and aromatic amine (**2**, 2.3 mmol) was heated for 5–10 minutes at 100 °C under solvent-free conditions to afford the thiourea derivative as an off-white solid. To the solid, chloroacetic acid (**3**, 6.9 mmol) was added, and the mixture was further heated for 10–15 minutes. After completion of the reaction as monitored by TLC, crushed ice was added to the reaction mixture, and the resulting solid was filtered, washed with water, and purified by filtration through a pad of silica gel using a 7:3 petroleum ether–ethylacetate mixture to afford the pure product **4**.

(*Z*)-3-Ethyl-2-(2-phenylcyclohex-2-enylimino)thiazolidin-4-one (4a)

Yield: 0.55 g (79%). White solid, mp 118–119 °C. ^{1}H NMR (300 MHz, $CDCl_3$) δ (ppm): 7.23–7.48 (m, 5H, ArH), 6.12 (br s, 1H, C=CH), 4.34 (br s, 1H, CHN), 3.78 (s, 2H), 3.56 (m, 2H, CH_2N), 2.26–2.34 (m, 2H, Alicyclic protons), 1.95–1.98 (m, 1H, Alicyclic protons), 1.76–1.80 (m, 2H, Alicyclic protons), 1.66–1.68 (m, 1H, Alicyclic protons), 0.88 (t, J = 7.2 Hz, 3H). ^{13}C NMR (75 MHz, $CDCl_3$) δ (ppm): 171.40, 150.45, 141.61, 139.47, 127.92,

127.75, 126.46, 126.23, 58.50, 37.98, 32.63, 30.27, 25.96, 19.23, 11.99. IR (KBr) ν_{max} 3028, 1712, 1628, 1483 cm^{-1}. Anal. calcd for: C, 67.97; H, 6.71; N, 9.32. Found: C, 67.83; H, 6.70; N, 9.30.

(*Z*)-3-Butyl-2-(2-phenylcyclohex-2-enylimino)thiazolidin-4-one (4b)

Yield: 0.64 g (84%). White solid, mp 150–151 °C. ^{1}H NMR (300 MHz, $CDCl_3$) δ (ppm): 7.13–7.29 (m, 5H, ArH), 6.09 (br s, 1H, C=CH), 4.50 (br s, 1H, CHN), 3.76 (s, 2H), 3.53 (m, 2H), 2.15–2.18 (m, 2H, Alicyclic protons), 1.95–2.01 (m, 1H, Alicyclic protons), 1.88–1.91 (m, 2H, Alicyclic protons), 1.67–1.77 (m, 1H, Alicyclic protons), 1.19 (m, 2H), 1.01 (m, 2H), 0.71(t, *J* = 6.9 Hz, 3H). ^{13}C NMR (75 MHz, $CDCl_3$) δ (ppm): 171.43, 150.53, 141.61, 139.57, 127.56, 127.58, 126.39, 126.17, 58.42, 42.62, 32.48, 30.12, 28.78, 25.90, 19.64, 19.31, 13.60. IR (KBr) ν_{max} 2986, 1705, 1630, 1463 cm^{-1}. Anal. calcd for: C, 69.47; H, 7.36; N, 8.53. Found: C, 69.55; H, 7.39; N, 8.56.

(*Z*)-3-Benzyl-2-(2-phenylcyclohex-2-enylimino)thiazolidin-4-one (4c)

Yield: 0.69 g (83%). White solid, mp 132–133 °C. ^{1}H NMR (300 MHz, $CDCl_3$) δ (ppm): 7.24–7.31 (m, 5H, ArH), 7.10–7.14 (m, 5H, ArH), 6.12 (br s, 1H, C=CH), 4.39 (br s, 1H, CHN), 4.85 (d, *J* = 14.1 Hz, 1H, benzylic), 4.60 (d, *J* = 14.1 Hz, 1H, benzylic), 3.73 (s, 2H), 2.12–2.27 (m, 2H, Alicyclic protons), 1.89–1.93 (m, 1H, Alicyclic protons), 1.73–1.77 (m, 2H, Alicyclic protons), 1.68–1.71 (m, 1H, Alicyclic protons). ^{13}C NMR (75 MHz, $CDCl_3$) δ (ppm): 171.23, 150.48, 141.75, 139.48, 136.18, 128.86, 128.13, 128.06, 127.91, 127.34, 126.58, 126.35, 58.19, 46.01, 32.44, 30.48, 26.01, 19.01. IR (KBr) ν_{max} 2925, 1712, 1628, 1427 cm^{-1}. Anal. calcd for: C, 72.89; H, 6.12; N, 7.73. Found: C, 72.95; H, 6.11; N, 7.70.

(*Z*)-3-Cyclohexyl-2-(2-phenylcyclohex-2-enylimino)thiazolidin-4-one (4d)

Yield: 0.69 g (84%). White solid, mp 139–140 °C. ^{1}H NMR (300 MHz, $CDCl_3$) δ (ppm): 7.21–7.26 (m, 5H, ArH), 6.10 (br s, 1H, C=CH), 4.32 (br s, 1H, CHN), 3.82 (s, 2H), 2.48 (m, 1H, CHN), 2.18–2.28 (m, 4H, Alicyclic protons), 1.96–1.98 (m, 4H, Alicyclic protons), 1.72–

1.76 (m, 4H, Alicyclic protons), 1.56–1.69 (m, 5H, Alicyclic protons), 0.81–086 (m, 1H, Alicyclic protons), 0.66–0.68 (m, 1H, Alicyclic protons). ^{13}C NMR (75 MHz, $CDCl_3$) δ (ppm): 171.86, 151.20, 141.91, 139.71, 128.70, 127.97, 127.81, 126.48, 126.37, 126.21, 29.19, 32.45, 29.99, 25.98, 25.80, 19.55, 6.78, 6.49. IR (KBr) ν_{max} 2979, 1710, 1634, 1481 cm^{-1}. Anal. calcd for: C, 71.15; H, 7.39; N, 7.90. Found: C, 71.26; H, 7.36; N, 7.94.

(*Z*)-3-Phenyl-2-(2-phenylcyclohex-2-enylimino)thiazolidin-4-one (4e)

Yield: 0.64 g (80%). White solid, mp 142–143 °C. ^{1}H NMR (300 MHz, $CDCl_3$) δ (ppm): 7.18–7.33 (m, 10H, ArH), 6.75 (br s, 1H, C=CH), 4.39 (br s, 1H, CHN), 3.95 (dd, *J* = 13.4, 5.4 Hz, 2H), 2.17–2.18 (m, 2H, Alicyclic protons), 1.93–2.00 (m, 1H, Alicyclic protons), 1.78–1.85 (m, 2H, Alicyclic protons), 1.61–1.66 (m, 1H, Alicyclic protons). ^{13}C NMR (75 MHz, $CDCl_3$) δ (ppm): 171.07, 150.86, 142.19, 139.85, 134.98, 128.70, 128.12, 127.93, 127.70, 127.56, 126.68, 126.41, 59.33, 32.68, 29.49, 25.75, 19.81. IR (KBr) ν_{max} 2931, 1727, 1639, 1516 cm^{-1}. Anal. calcd for: C, 72.38; H, 5.79; N, 8.04. Found: C, 72.49; H, 5.77; N, 8.06.

(*Z*)-2-(2-Phenylcyclohex-2-enylimino)-3-*p*-tolylthiazolidin-4-one (4f)

Yield: 0.69 g (82%). White solid, mp 148–149 °C. ^{1}H NMR (300 MHz, $CDCl_3$) δ (ppm): 7.18–7.33 (m, 5H, ArH), 7.01 (d, *J* = 7.8 Hz, 2H), 6.61 (d, *J* = 7.2 Hz, 2H), 5.95 (br s, 1H, C=CH), 4.39 (br s, 1H, CHN), 3.93 (dd, *J* = 13.4, 5.4 Hz, 2H), 2.28 (s, 3H, CH_3), 2.16–2.17 (m, 2H, Alicyclic protons), 1.94–1.96 (m, 1H, Alicyclic protons), 1.78–1.80 (m, 2H, Alicyclic protons), 1.62–1.66 (m, 1H, Alicyclic protons). ^{13}C NMR (75 MHz, $CDCl_3$) δ (ppm): 171.12, 150.91, 142.22, 139.92, 137.96, 132.46, 129.41, 127.89, 127.51, 127.41, 126.72, 126.38, 59.38, 32.62, 29.59, 25.79, 21.13, 19.83. IR (KBr) ν_{max} 2946, 1726, 1632, 1512 cm^{-1}. Anal. calcd for: C, 72.89; H, 6.12; N, 7.73. Found: C, 73.02; H, 6.11; N, 7.76.

(*Z*)-3-(4-Fluorophenyl)-2-(2-phenylcyclohex-2-enylimino)thiazolidin-4-one (4g)

Yield: 0.66 g (80%). White solid, mp 161–162 °C. ^{1}H NMR (300 MHz, $CDCl_3$) δ (ppm): 7.19–7.33 (m, 5H, ArH), 6.85–6.91 (m, 2H), 6.65–6.70 (m, 2H), 5.95 (br s, 1H, C=CH), 4.39 (br s, 1H, CHN), 3.93 (dd, *J* = 13.4, 5.4 Hz, 2H), 2.17–2.19 (m, 2H, Alicyclic protons), 1.83–1.99 (m, 1H, Alicyclic protons), 1.72–1.74 (m, 2H, Alicyclic protons), 1.56–1.68 (m, 1H, Alicyclic protons). ^{13}C NMR (75 MHz, $CDCl_3$) δ (ppm): 170.99, 163.52, 160.24, 150.86, 142.21, 139.83, 130.74, 130.70, 129.53, 129.41, 127.98, 127.64, 126.66, 126.44, 115.85, 115.55, 59.29, 32.62, 29.40, 25.74, 19.89. IR (KBr) ν_{max} 2924, 1720, 1621, 1498 cm^{-1}. Anal. calcd for: C, 68.83; H, 5.23; N, 7.64. Found: C, 68.90; H, 5.21; N, 7.61.

(*Z*)-3-(4-Chlorophenyl)-2-(2-phenylcyclohex-2-enylimino)thiazolidin-4-one (4h)

Yield: 0.73 g (83%). White solid, mp 156–157 °C. ^{1}H NMR (300 MHz, $CDCl_3$) δ (ppm): 7.24–7.34 (m, 5H, ArH), 7.18 (d, *J* = 7.2 Hz, 2H), 6.63 (d, *J* = 8.7 Hz, 2H), 5.96 (br s, 1H, C=CH), 4.39 (br s, 1H, CHN), 3.94 (dd, *J* = 13.4, 5.4 Hz, 2H), 2.17–2.18 (m, 2H, Alicyclic protons), 1.93–2.00 (m, 1H, Alicyclic protons), 1.78–1.85 (m, 2H, Alicyclic protons), 1.61–1.66 (m, 1H, Alicyclic protons). ^{13}C NMR (75 MHz, $CDCl_3$) δ (ppm): 170.70, 150.47, 142.22, 139.82, 133.86, 133.40, 129.02, 128.88, 127.97, 127.62, 126.68, 126.45, 59.30, 32.63, 29.48, 25.74, 19.82. IR (KBr) ν_{max} 2919, 1730, 1620, 1490 cm^{-1}. Anal. calcd for: C, 65.87; H, 5.00; N, 7.32. Found: C, 66.01; H, 5.03; N, 7.35.

(*Z*)-3-(4-Methoxyphenyl)-2-(2-phenylcyclohex-2-enylimino)thiazolidin-4-one (4i)

Yield: 0.72 g (82%). White solid, mp 181–183 °C. ^{1}H NMR (300 MHz, $CDCl_3$) δ (ppm 7.18–7.31 (m, 5H, ArH), 6.74 (d, *J* = 7.2 Hz, 2H), 6.63 (d, *J* = 9 Hz, 2H), 5.95 (br s, 1H, C=CH), 4.39 (br s, 1H, CHN), 3.92 (dd, *J* = 13.4, 5.4 Hz, 2H), 3.74 (s, 3H, OCH_3), 2.15–2.17 (m, 2H, Alicyclic protons), 1.92–2.00 (m, 1H, Alicyclic protons), 1.72–1.83 (m, 2H, Alicyclic protons), 1.58–1.66 (m, 1H, Alicyclic protons). ^{13}C NMR (75 MHz, $CDCl_3$) δ (ppm): 171.32, 159.04, 151.23, 142.20, 139.89, 128.73, 127.94, 127.58, 126.68, 126.41, 114.09, 59.33,

55.33, 32.61, 29.46, 25.78, 19.91. IR (KBr) ν_{max} 2952, 1730, 1639, 1519 cm^{-1}. Anal. calcd for: C, 69.81; H, 5.86; N, 7.40. Found: C, 69.77; H, 5.85; N, 7.43.

(*Z*)-3-(4-Bromophenyl)-2-(2-phenylcyclohex-2-enylimino)thiazolidin-4-one (4j)

Yield: 0.79 g (81%). White solid, mp 172–173 °C. ^{1}H NMR (300 MHz, $CDCl_3$) δ (ppm): 7.24–7.46 (m, 5H, ArH), 7.21 (d, *J* = 7.5 Hz, 2H), 6.56 (d, *J* = 8.7 Hz, 2H), 5.97 (br s, 1H, C=CH), 4.39 (br s, 1H, CHN), 3.96 (dd, *J* = 13.4, 5.4 Hz, 2H), 2.17–2.18 (m, 2H, Alicyclic protons), 1.93–2.00 (m, 1H, Alicyclic protons), 1.78–1.85 (m, 2H, Alicyclic protons), 1.61–1.66 (m, 1H, Alicyclic protons). ^{13}C NMR (75 MHz, $CDCl_3$) δ (ppm): 170.58, 150.35, 142.14, 139.74, 133.86, 131.79, 129.28, 127.91, 127.58, 126.61, 126.39, 121.94, 59.24, 32.58, 29.40, 25.67, 19.75. IR (KBr) ν_{max} 2917, 1719, 1626, 1473 cm^{-1}. Anal. calcd for: C, 59.02; H, 4.48; N, 6.56. Found: C, 59.11; H, 4.48; N, 6.52.

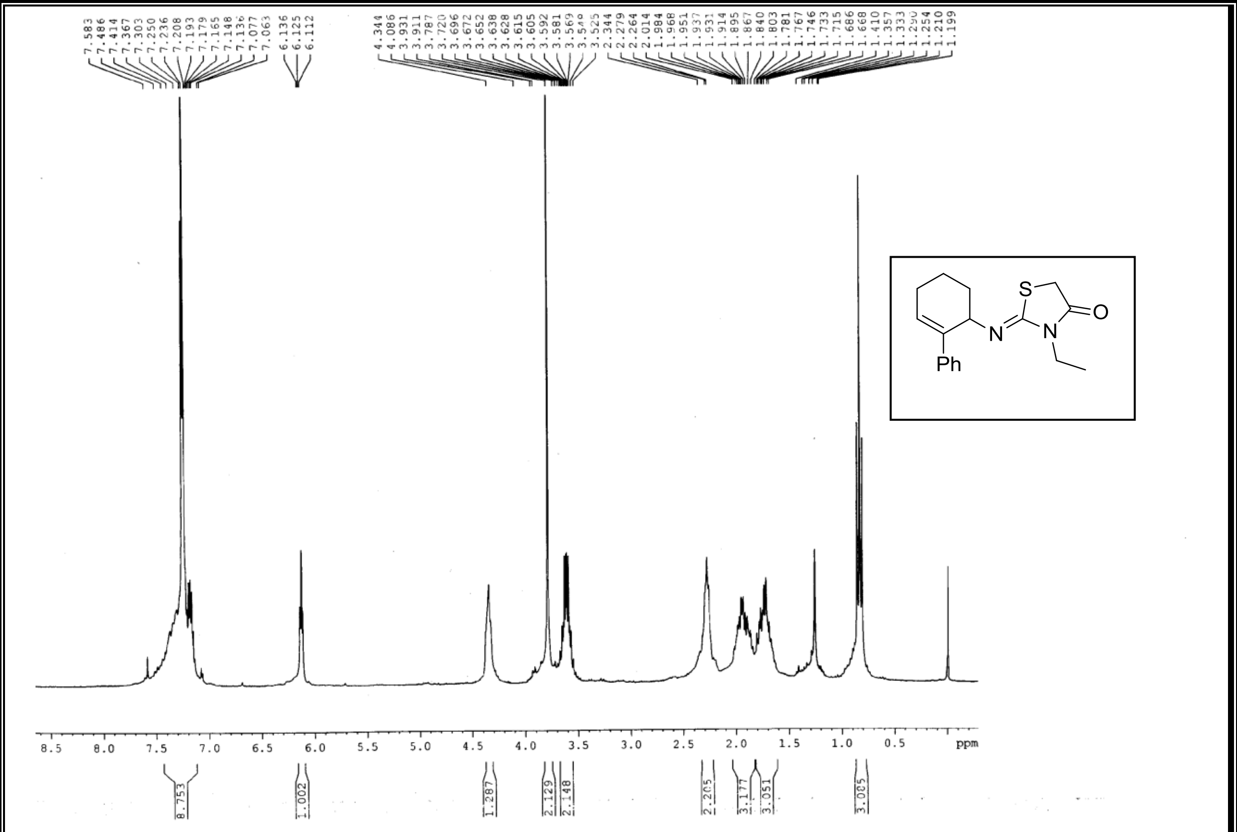

(*Z*)-3-Ethyl-2-(2-phenylcyclohex-2-enylimino)thiazolidin-4-one (4a)

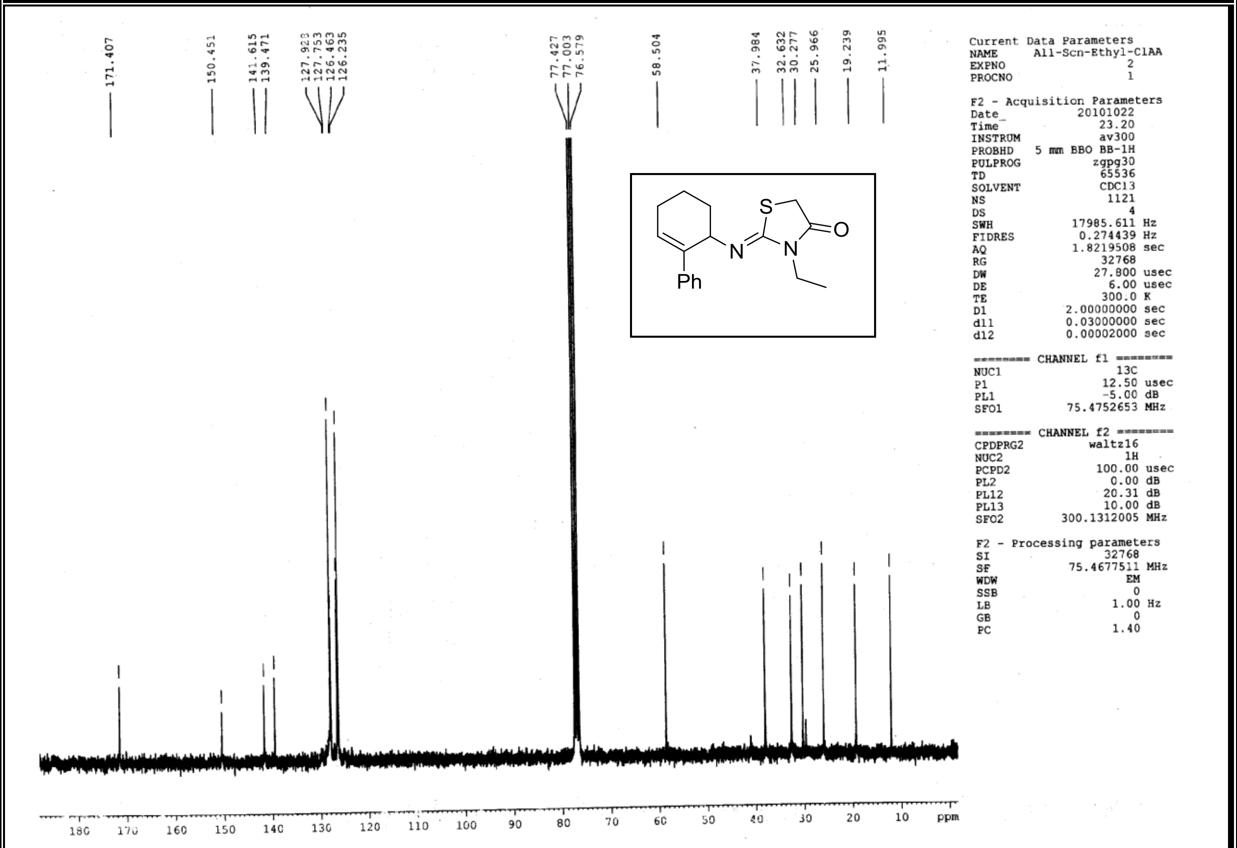

(*Z*)-3-Ethyl-2-(2-phenylcyclohex-2-enylimino)thiazolidin-4-one (4a)

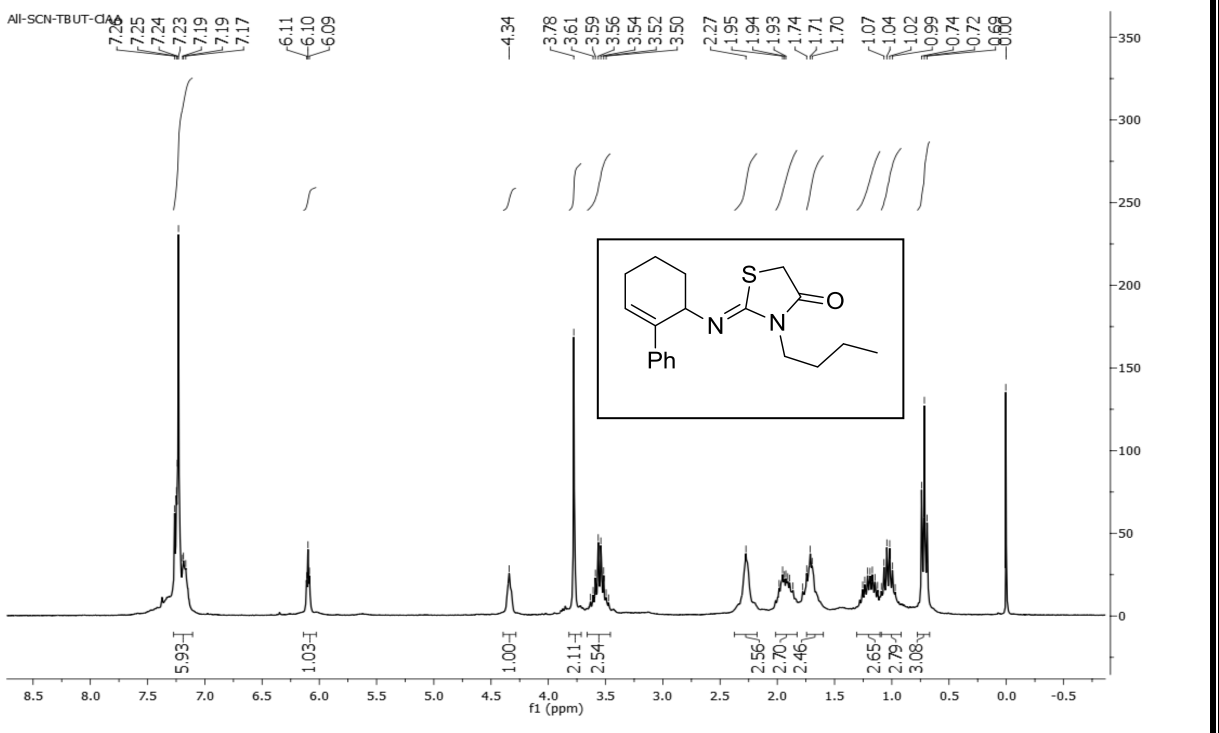

(*Z*)-3-Butyl-2-(2-phenylcyclohex-2-enylimino)thiazolidin-4-one (4b)

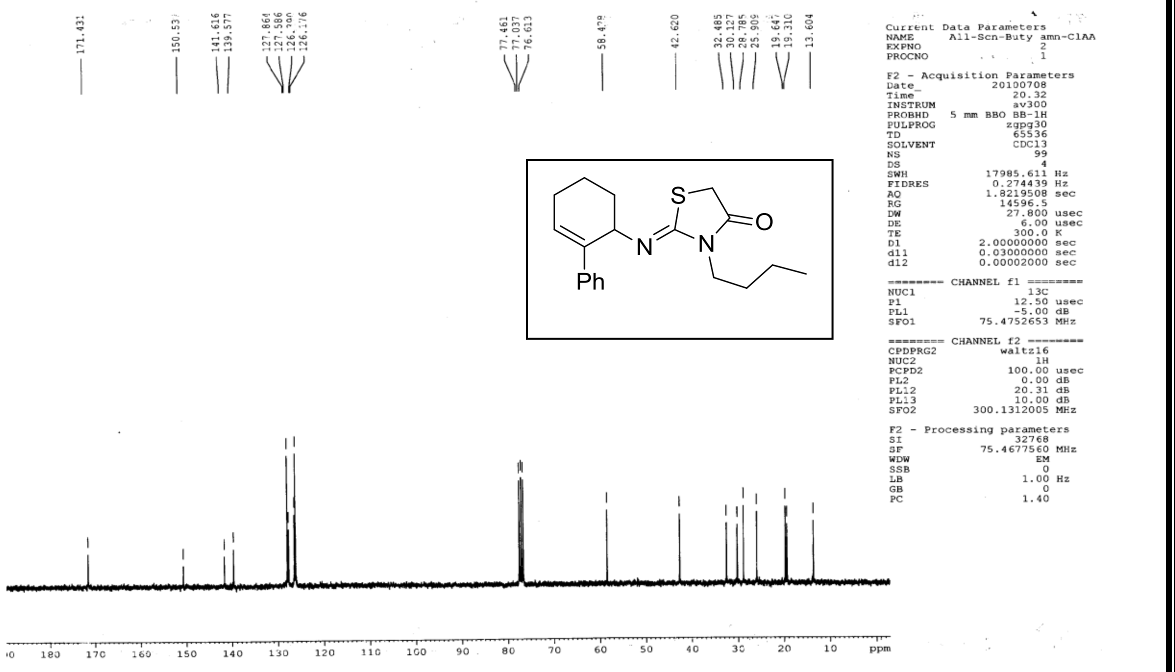

(*Z*)-3-Butyl-2-(2-phenylcyclohex-2-enylimino)thiazolidin-4-one (4b)

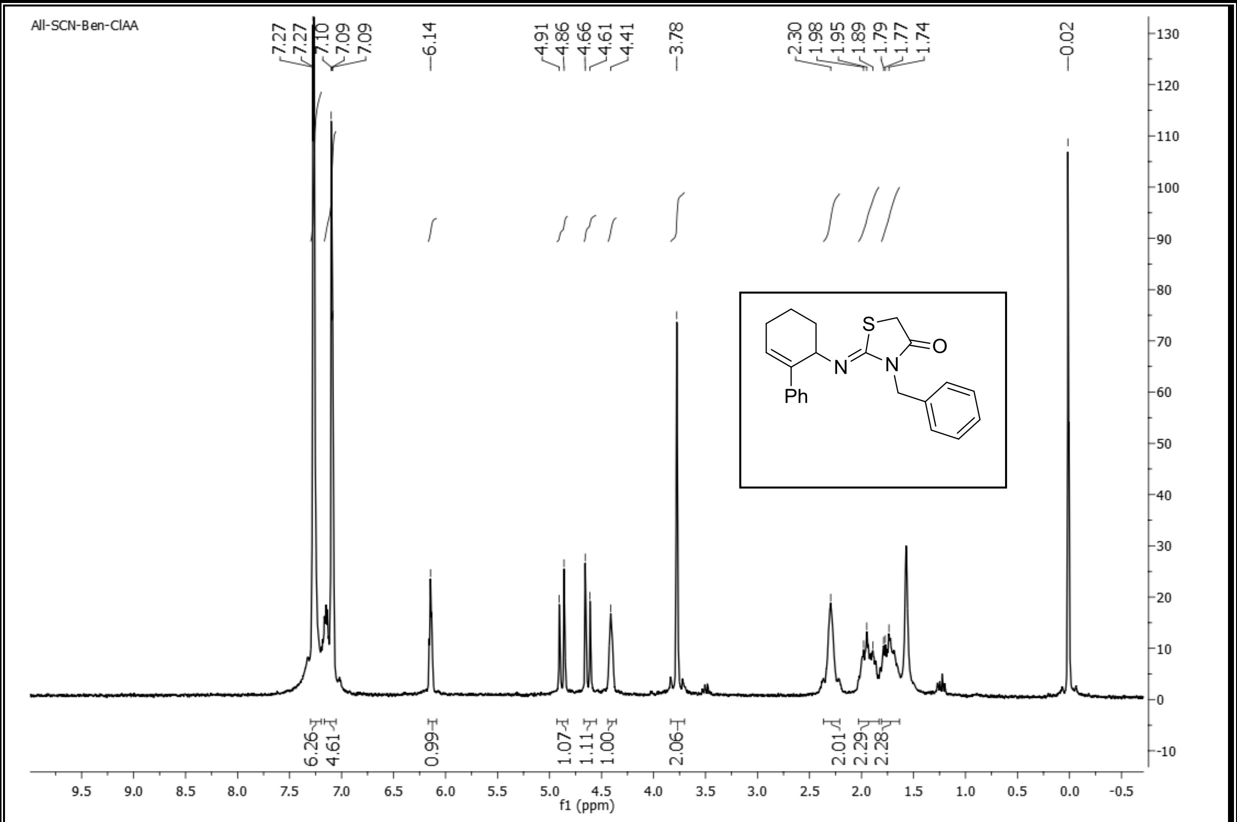

(*Z*)-3-Benzyl-2-(2-phenylcyclohex-2-enylimino)thiazolidin-4-one (4c)

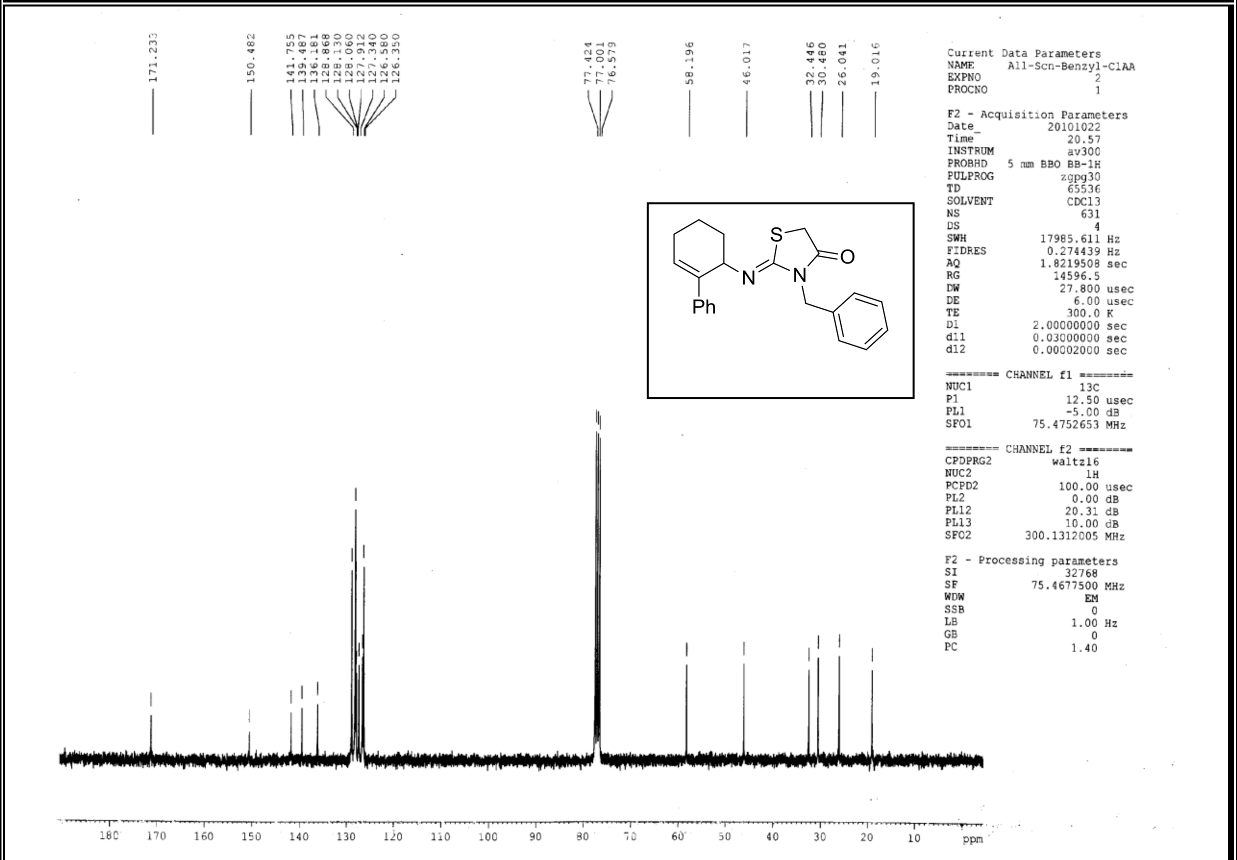

(*Z*)-3-Benzyl-2-(2-phenylcyclohex-2-enylimino)thiazolidin-4-one (4c)

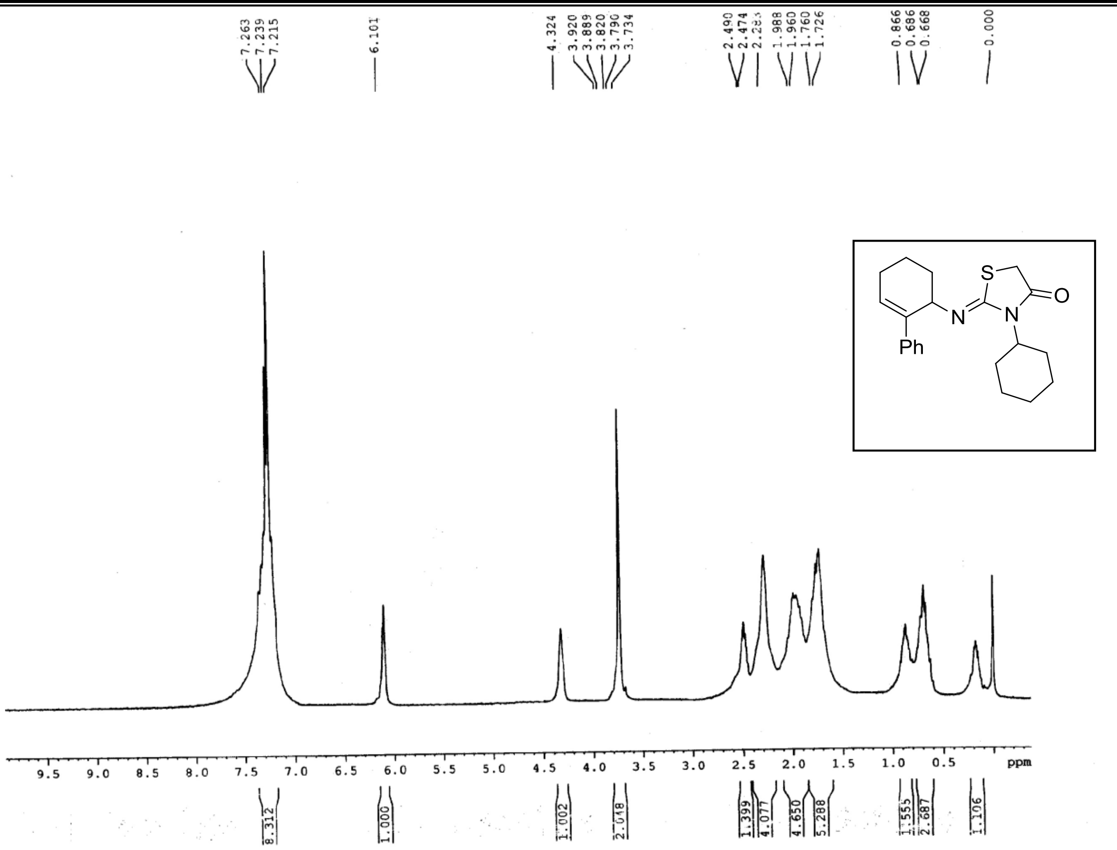

(*Z*)-3-Cyclohexyl-2-(2-phenylcyclohex-2-enylimino)thiazolidin-4-one (4d)

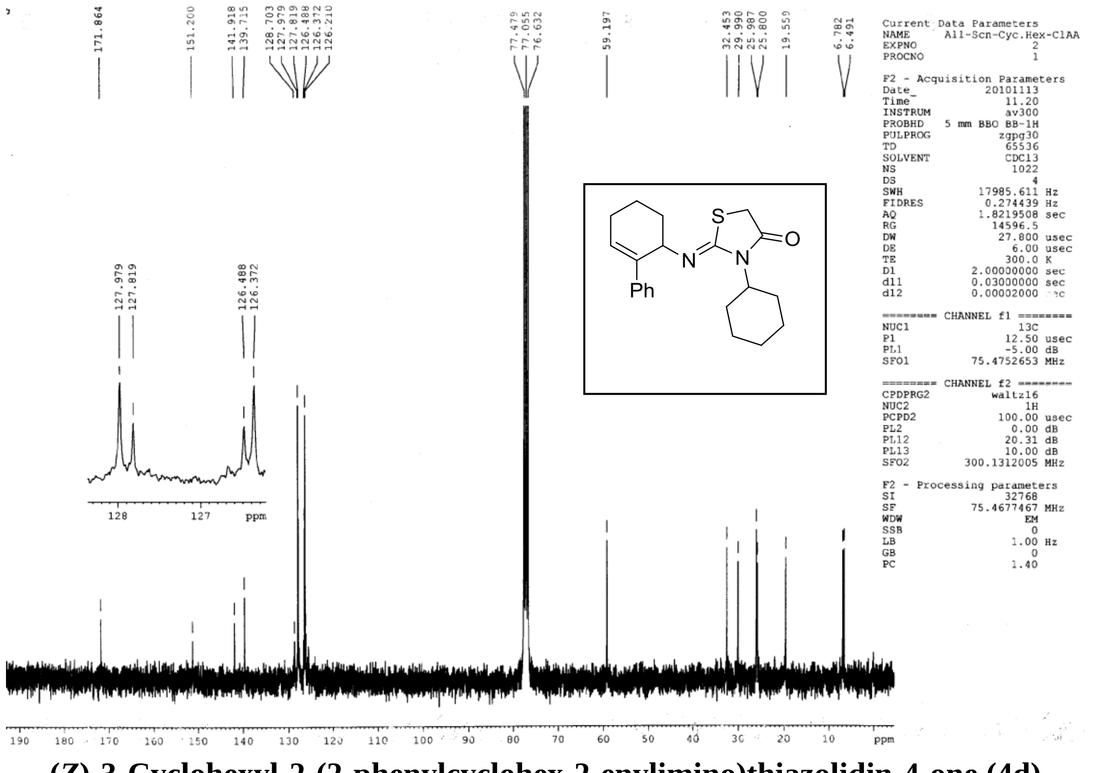

(*Z*)-3-Cyclohexyl-2-(2-phenylcyclohex-2-enylimino)thiazolidin-4-one (4d)

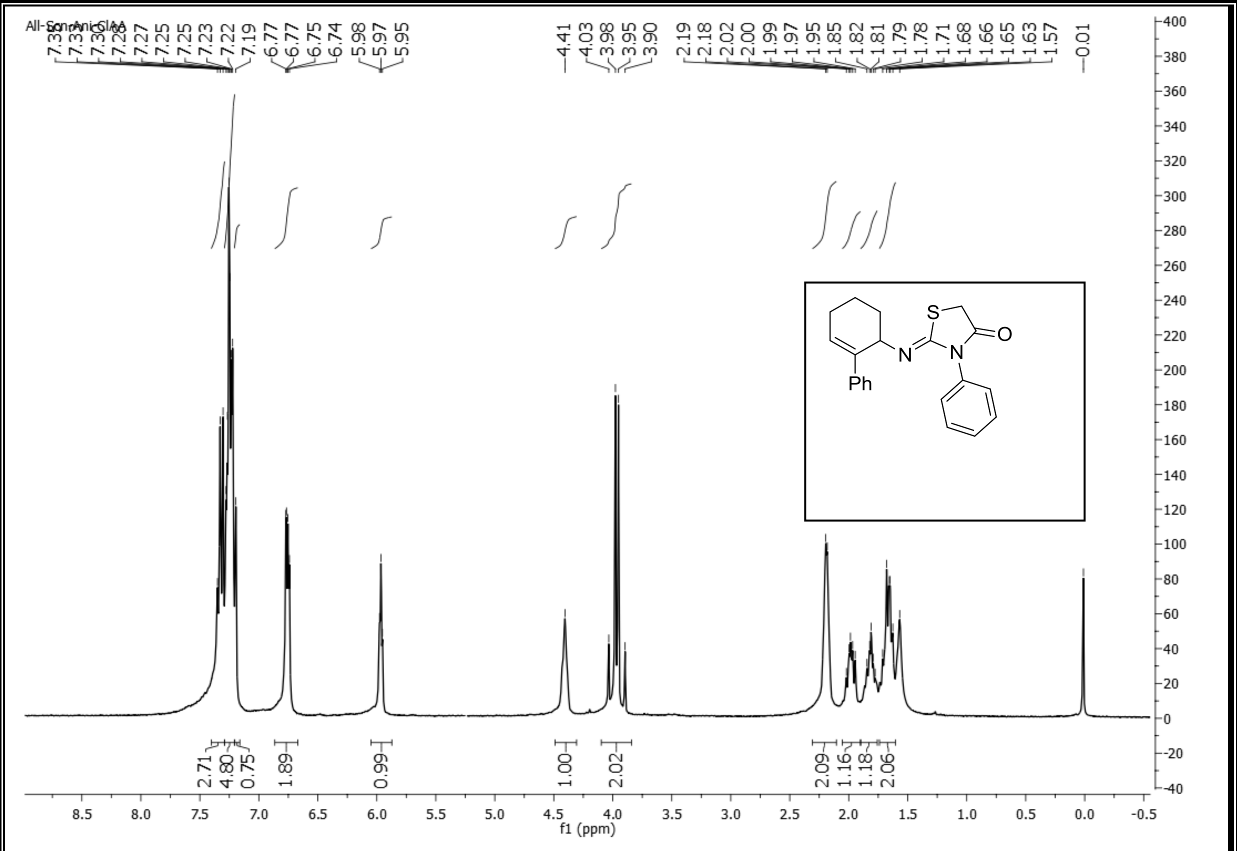

(*Z*)-3-Phenyl-2-(2-phenylcyclohex-2-enylimino)thiazolidin-4-one (4e)

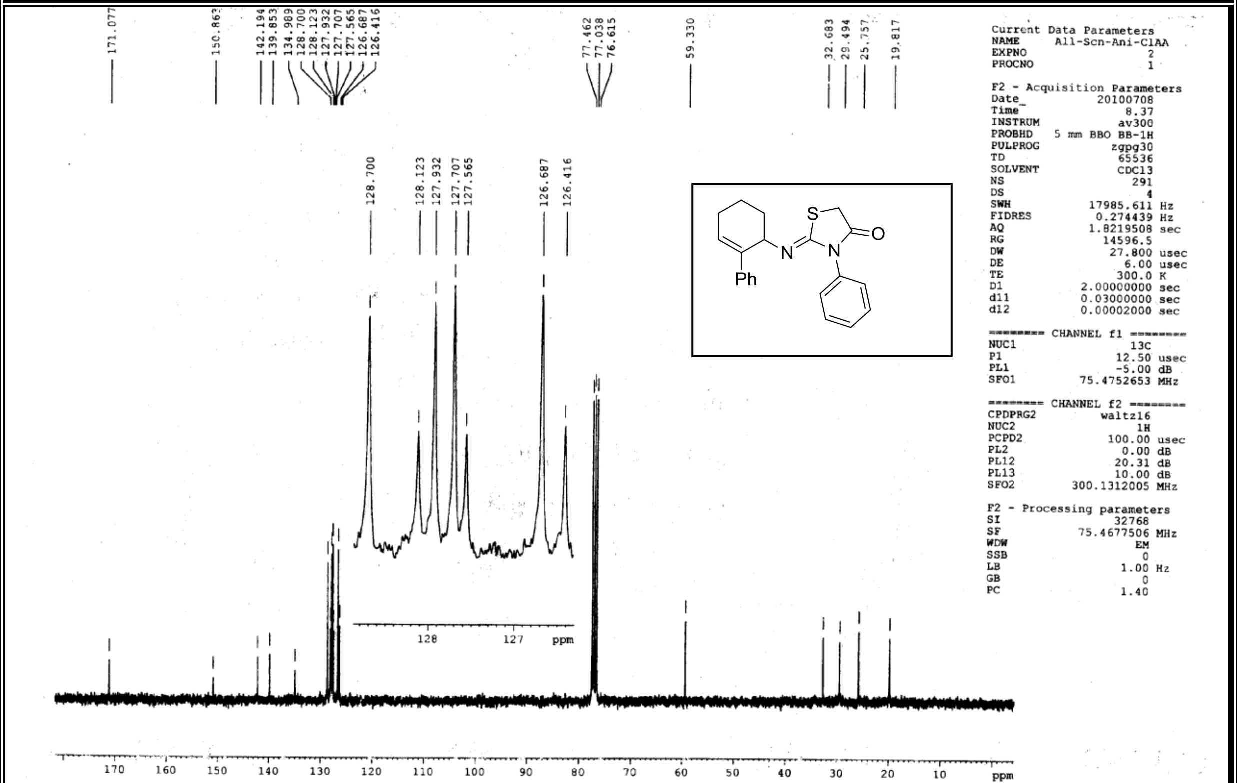

(*Z*)-3-Phenyl-2-(2-phenylcyclohex-2-enylimino)thiazolidin-4-one (4e)

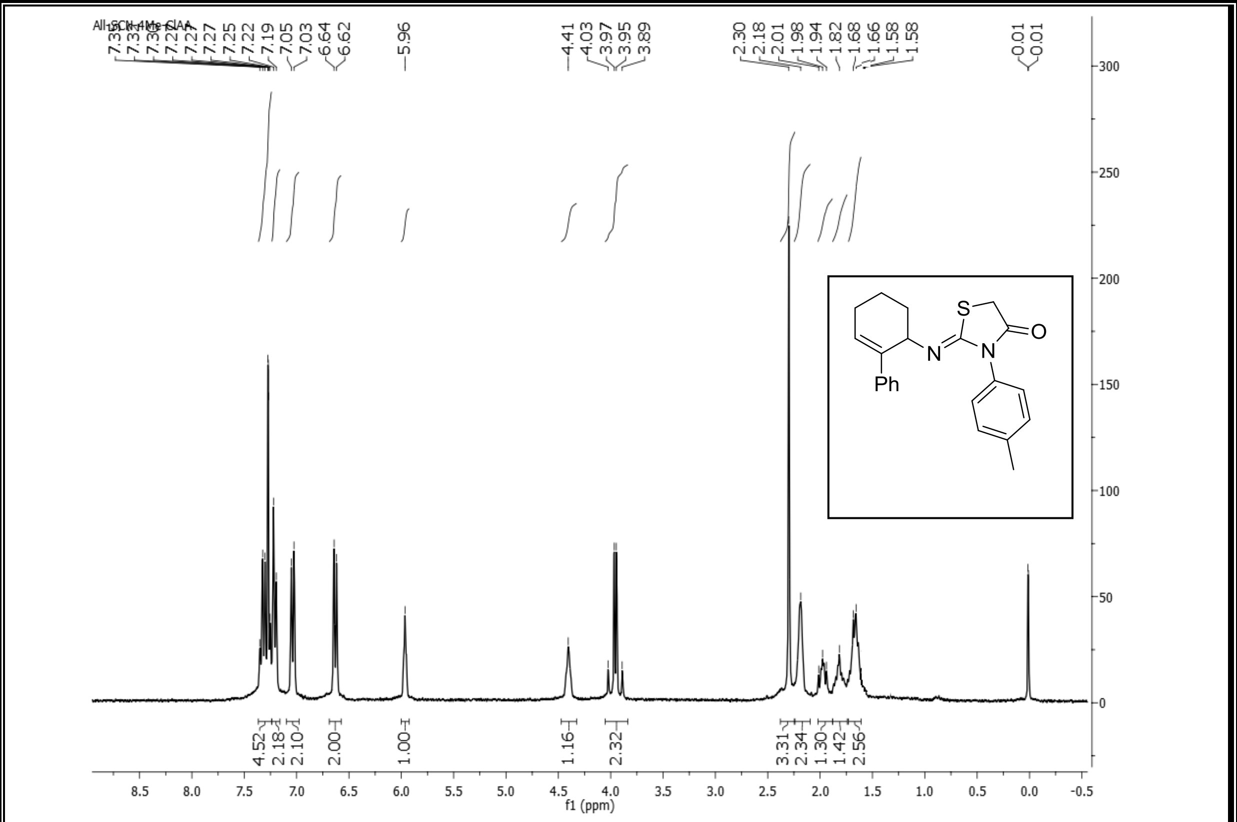

(*Z*)-2-(2-Phenylcyclohex-2-enylimino)-3-*p*-tolylthiazolidin-4-one (4f)

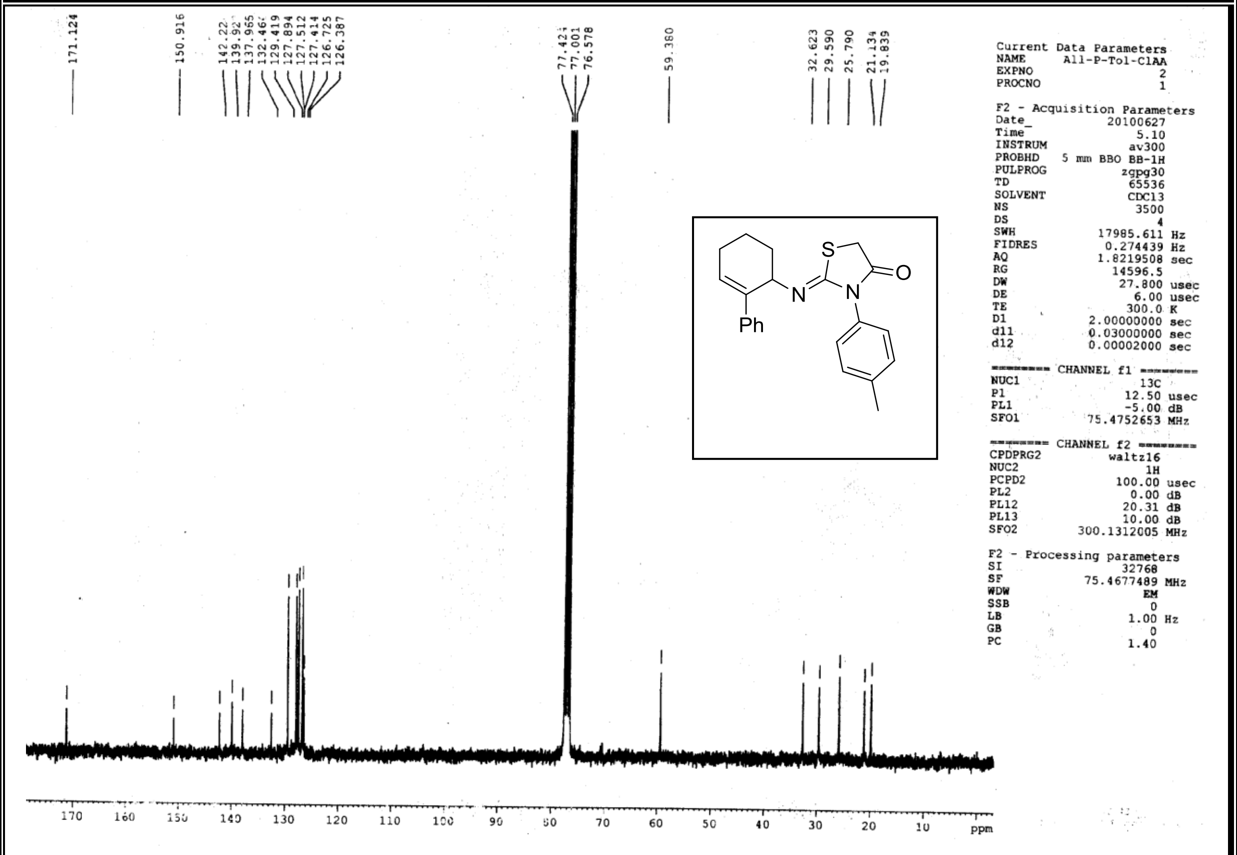

(*Z*)-2-(2-Phenylcyclohex-2-enylimino)-3-*p*-tolylthiazolidin-4-one (4f)

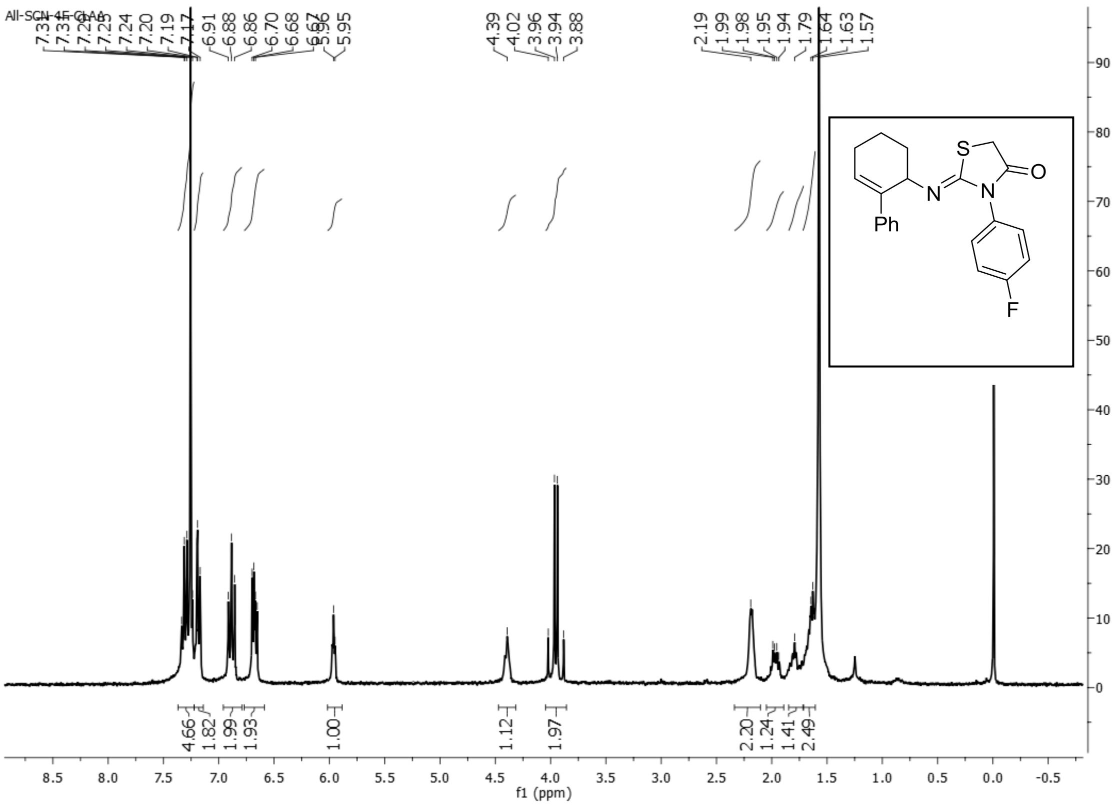

(*Z*)-3-(4-Fluorophenyl)-2-(2-phenylcyclohex-2-enylimino)thiazolidin-4-one (4g)

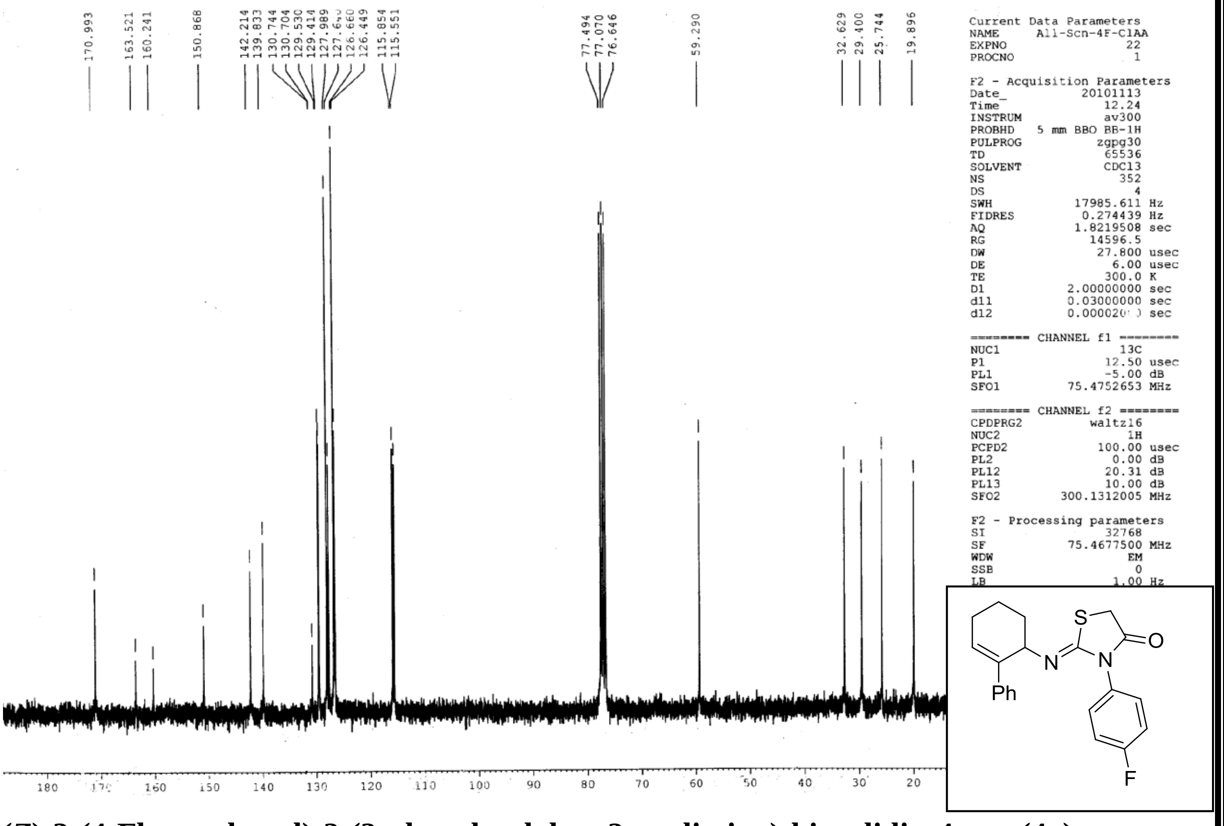

(*Z*)-3-(4-Fluorophenyl)-2-(2-phenylcyclohex-2-enylimino)thiazolidin-4-one (4g)

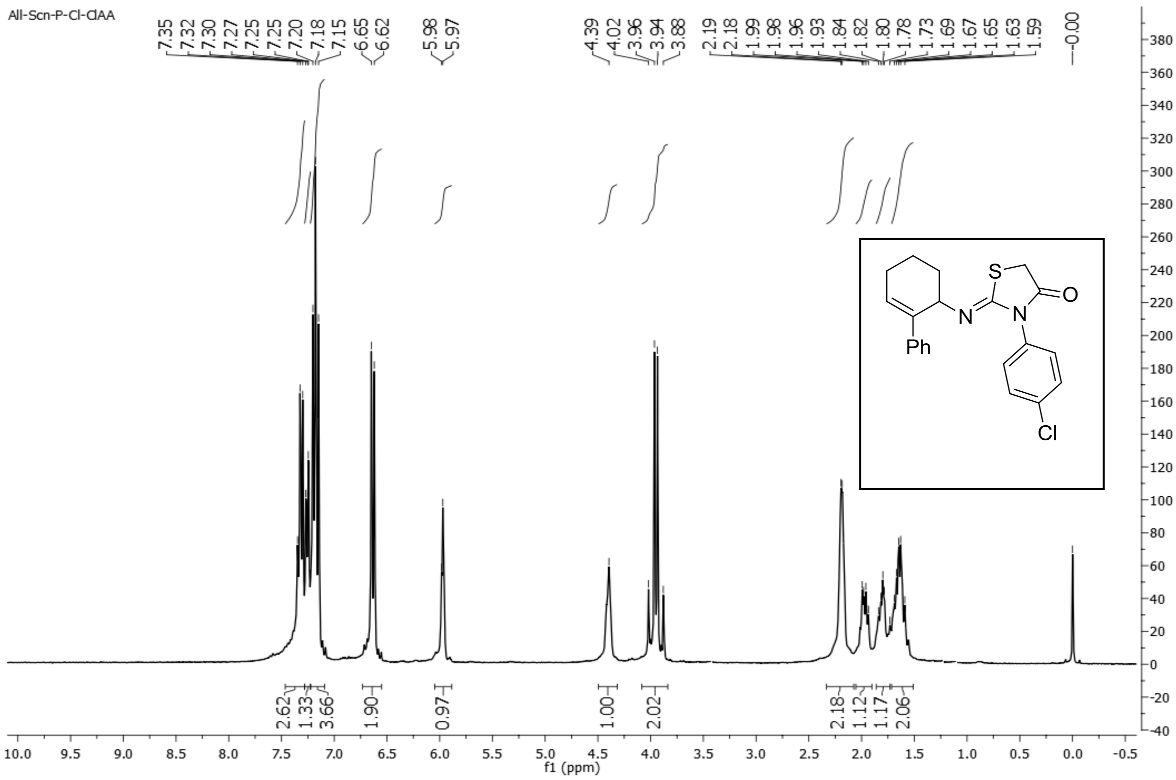

(*Z*)-3-(4-Chlorophenyl)-2-(2-phenylcyclohex-2-enylimino)thiazolidin-4-one (4h)

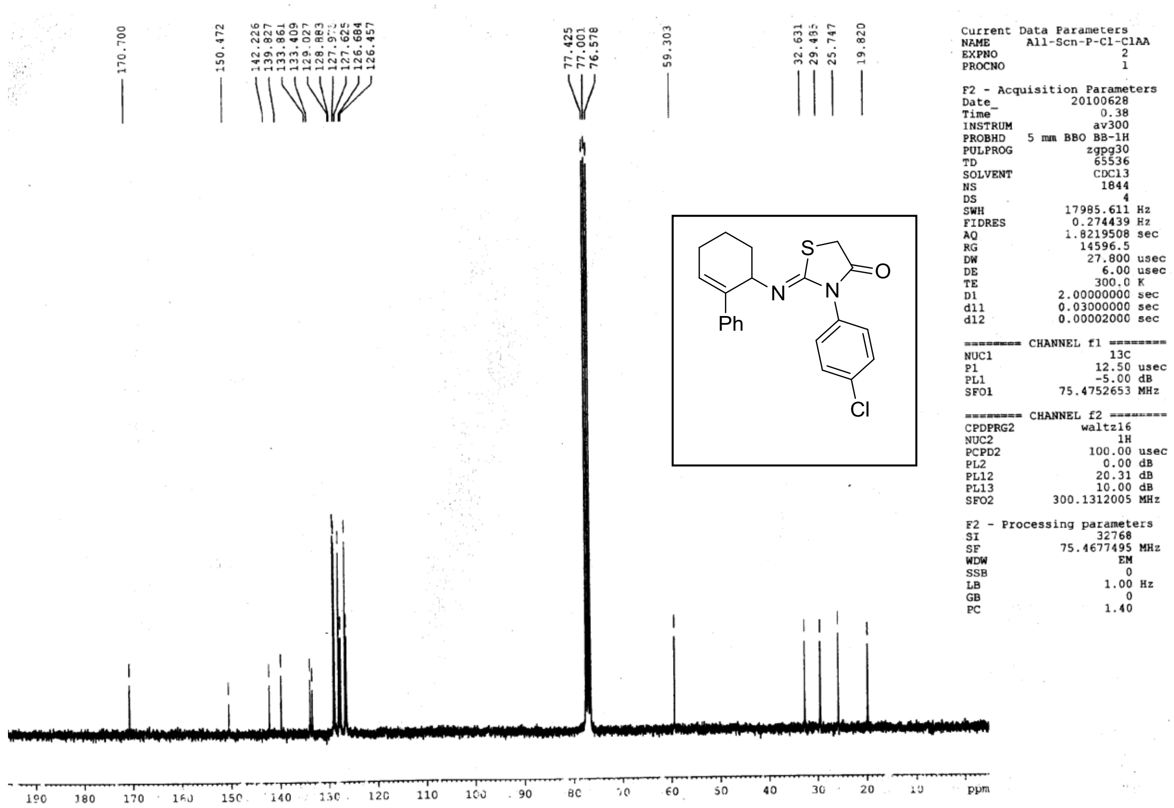

(*Z*)-3-(4-Chlorophenyl)-2-(2-phenylcyclohex-2-enylimino)thiazolidin-4-one (4h)

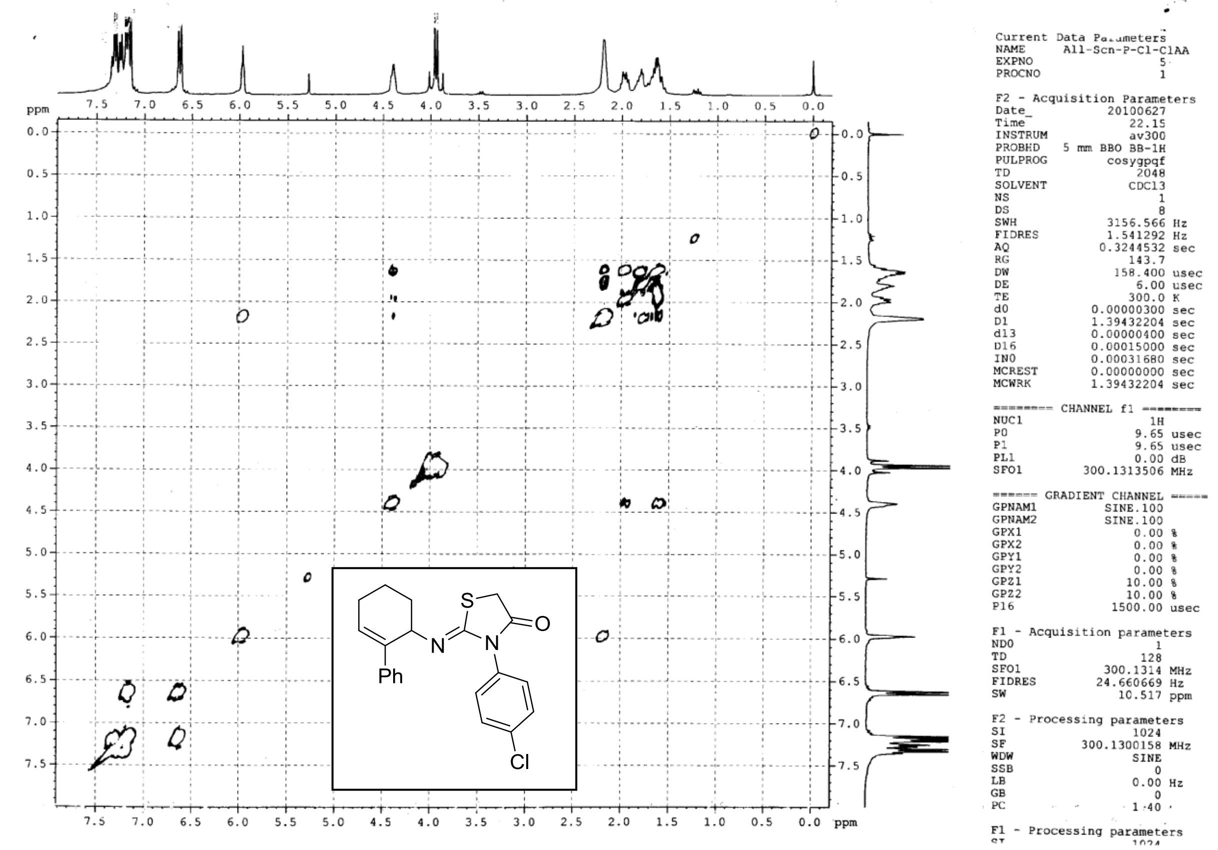

(*Z*)-3-(4-Chlorophenyl)-2-(2-phenylcyclohex-2-enylimino)thiazolidin-4-one (4h)

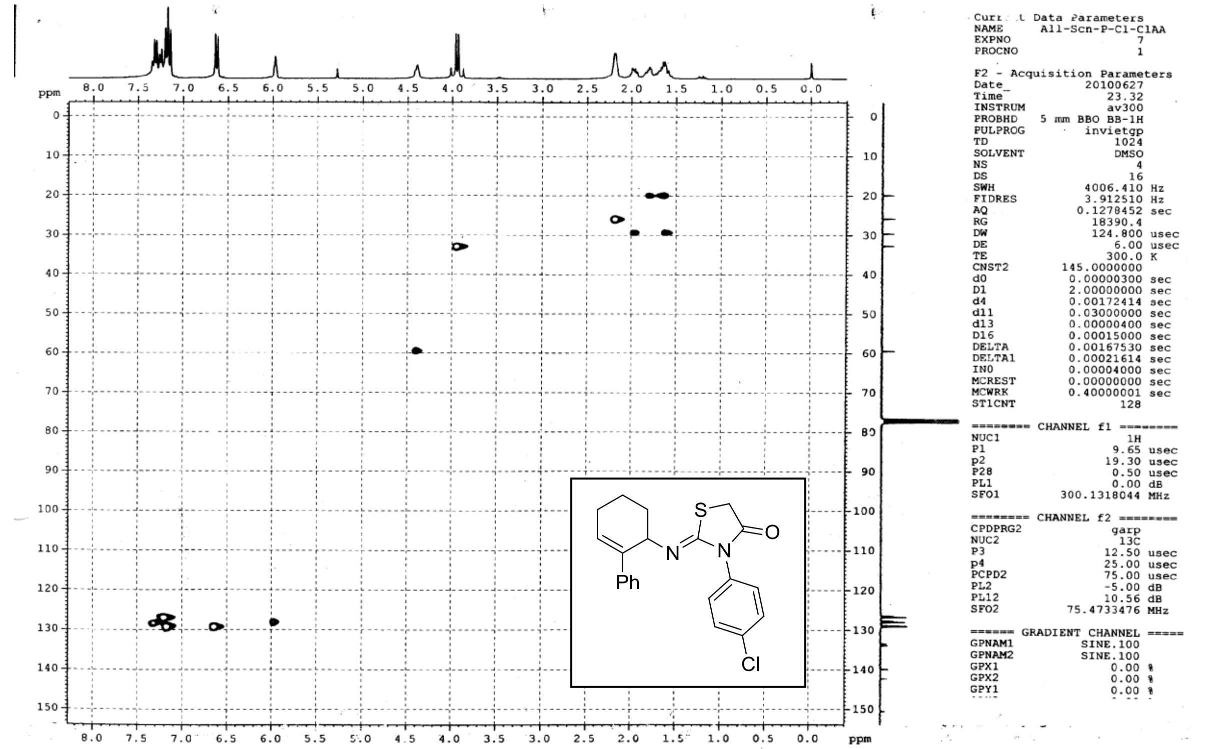

(*Z*)-3-(4-Chlorophenyl)-2-(2-phenylcyclohex-2-enylimino)thiazolidin-4-one (4h)

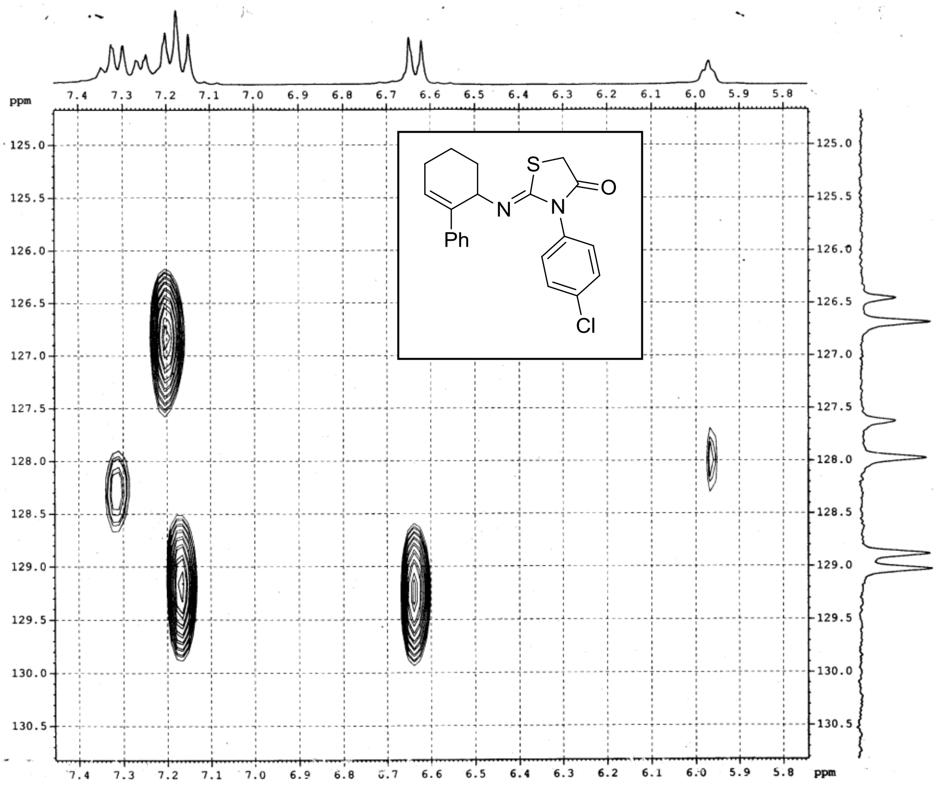

(*Z*)-3-(4-Chlorophenyl)-2-(2-phenylcyclohex-2-enylimino)thiazolidin-4-one (4h)

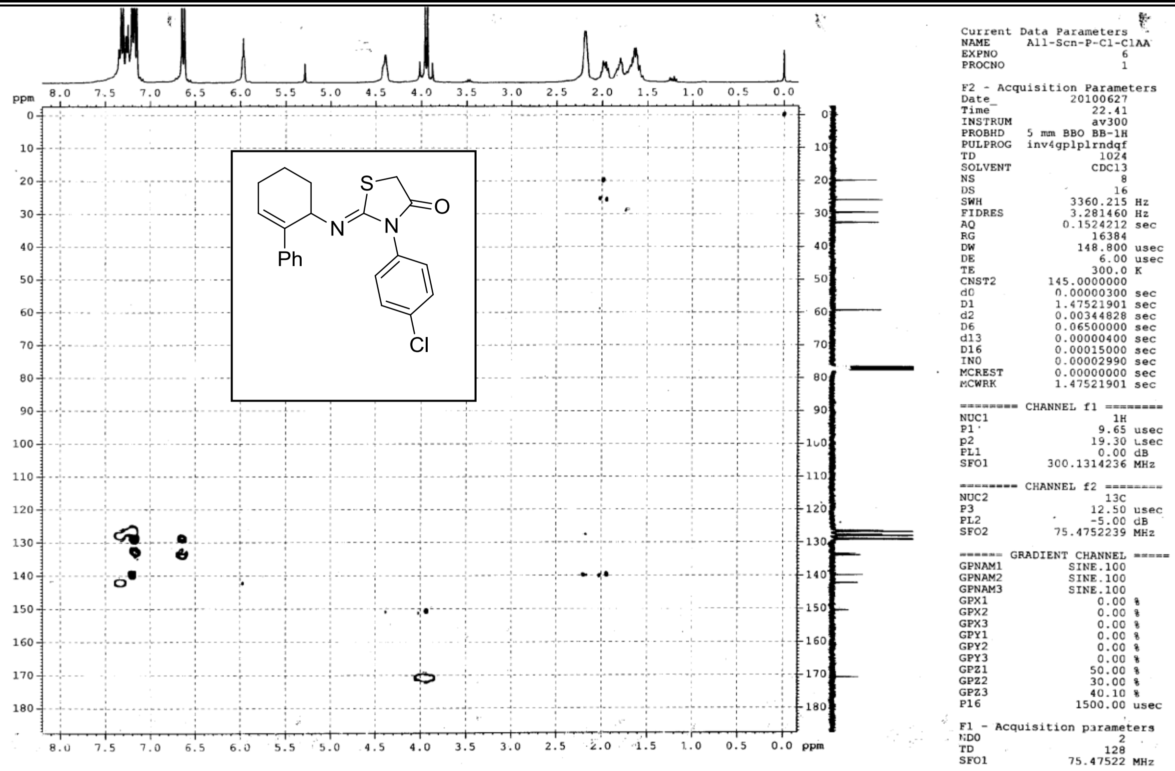

(*Z*)-3-(4-Chlorophenyl)-2-(2-phenylcyclohex-2-enylimino)thiazolidin-4-one (4h)

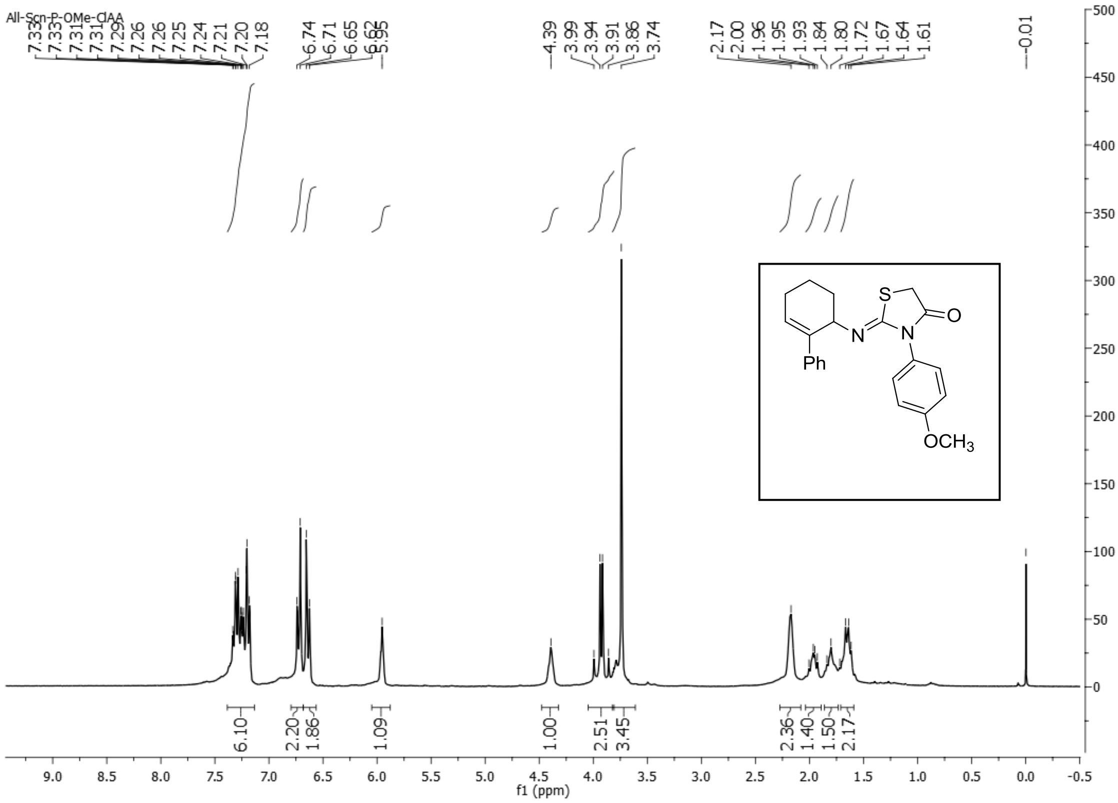

(*Z*)-3-(4-Methoxyphenyl)-2-(2-phenylcyclohex-2-enylimino)thiazolidin-4-one (4i)

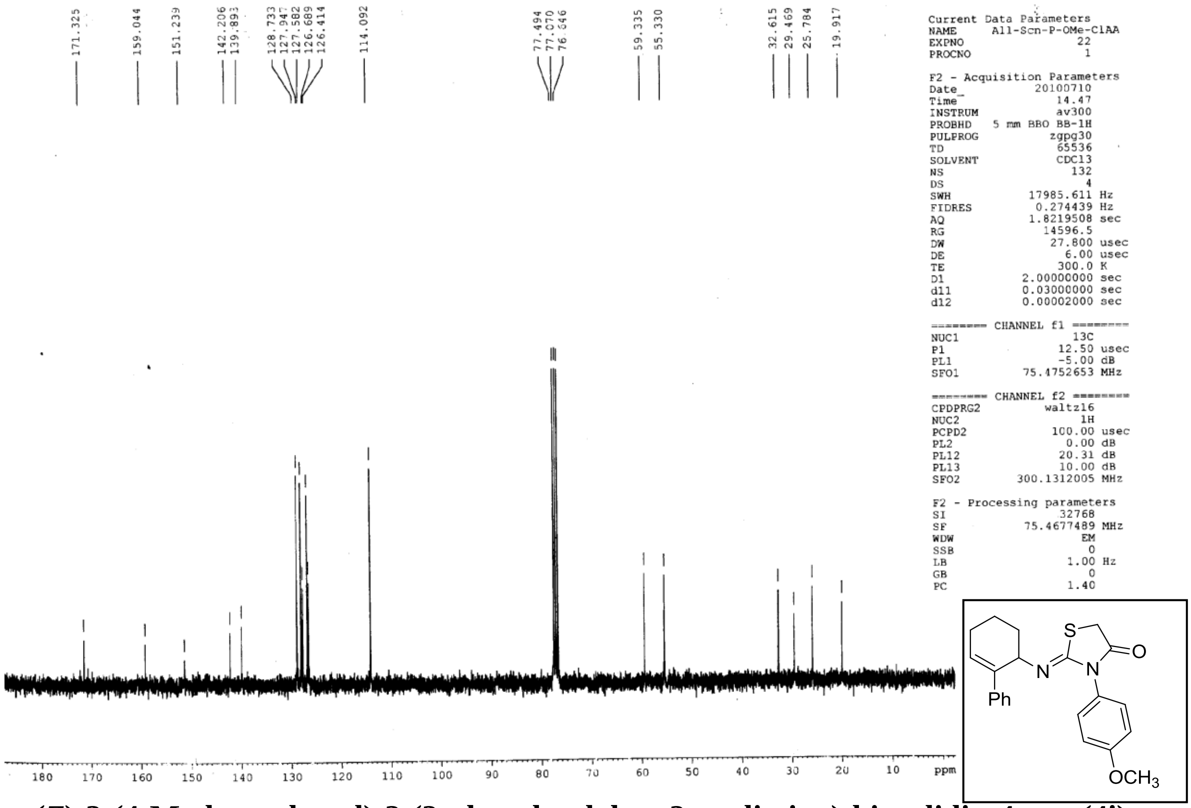

(*Z*)-3-(4-Methoxyphenyl)-2-(2-phenylcyclohex-2-enylimino)thiazolidin-4-one (4i)

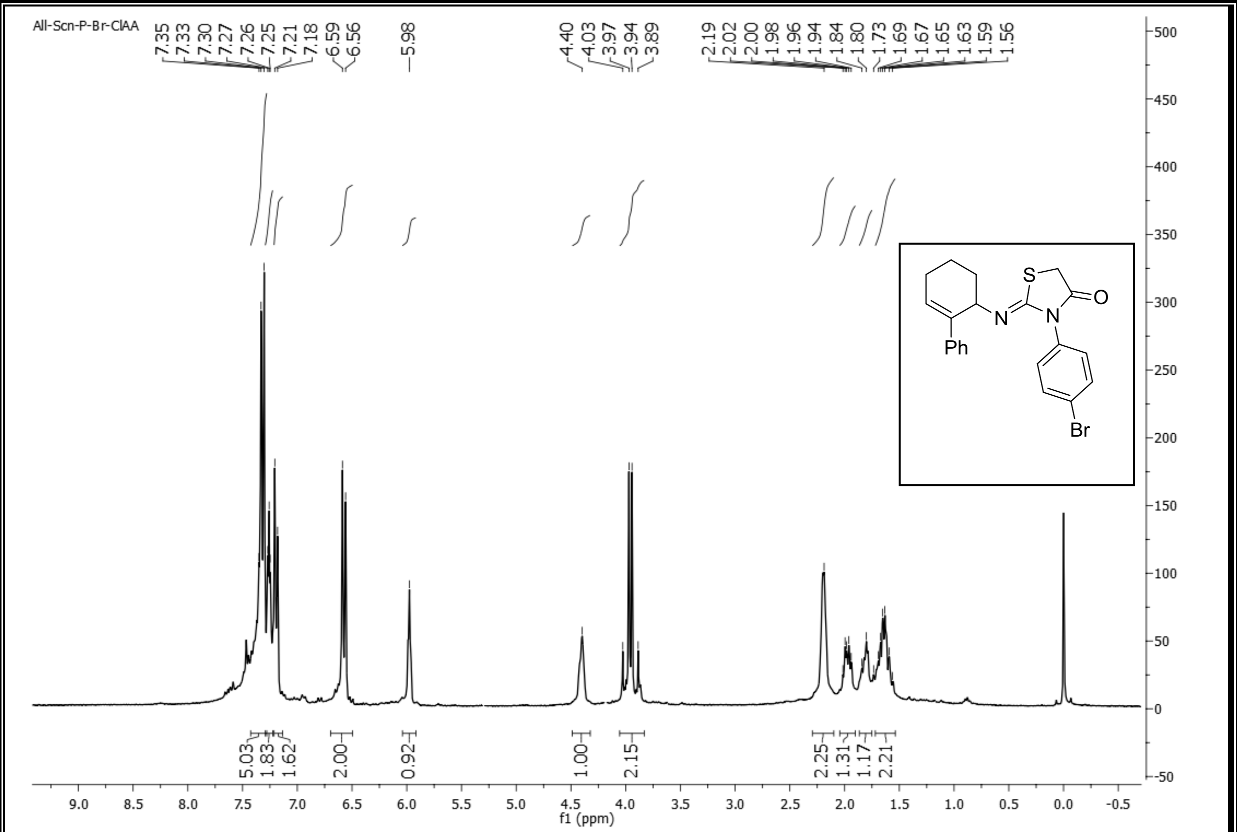

(*Z*)-3-(4-Bromophenyl)-2-(2-phenylcyclohex-2-enylimino)thiazolidin-4-one (4j)

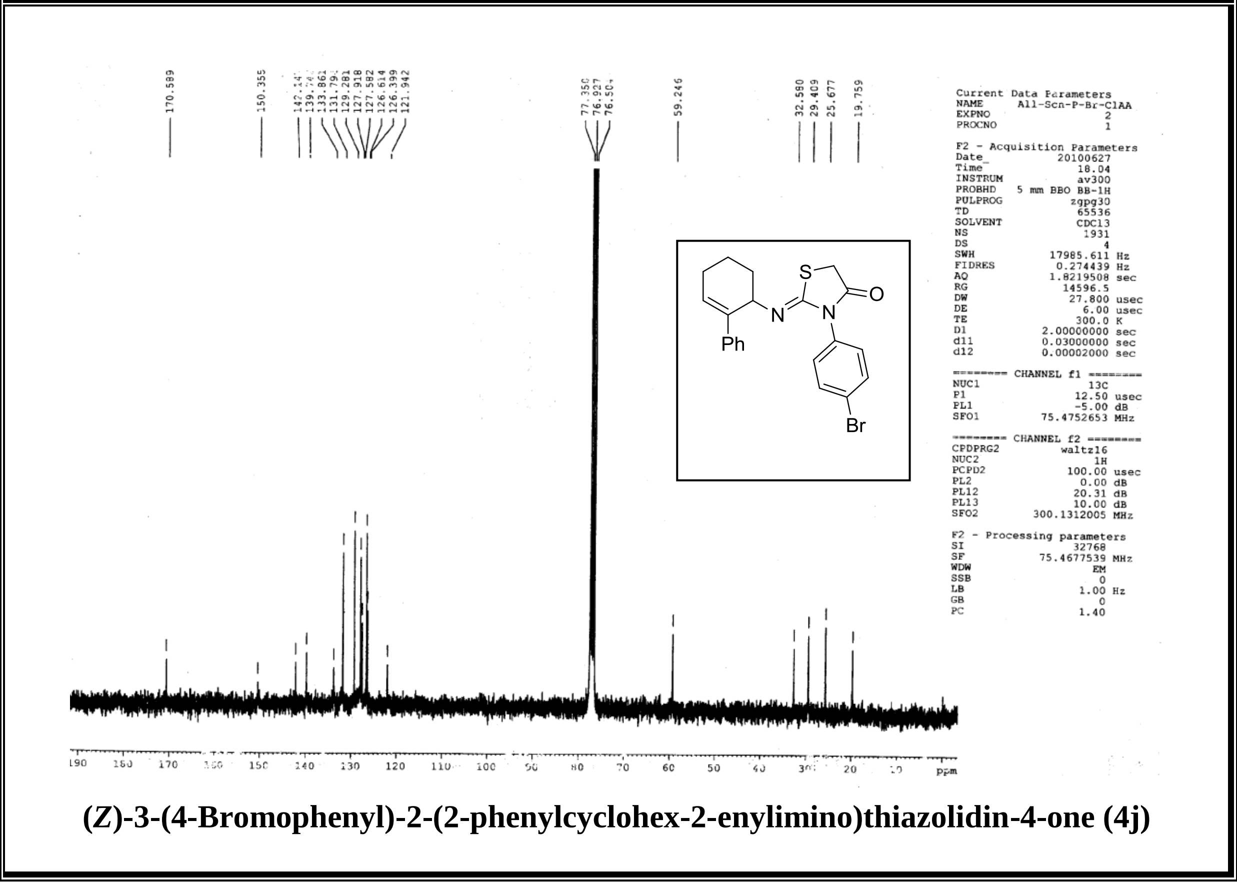

(*Z*)-3-(4-Bromophenyl)-2-(2-phenylcyclohex-2-enylimino)thiazolidin-4-one (4j)
